# Quorum Sensing Inhibition Selects for Virulence and Cooperation in *Pseudomonas aeruginosa*

**DOI:** 10.1371/journal.ppat.1000883

**Published:** 2010-05-06

**Authors:** Thilo Köhler, Gabriel G. Perron, Angus Buckling, Christian van Delden

**Affiliations:** 1 Service of Infectious Diseases, University Hospital Geneva and Department of Microbiology and Molecular Medicine, University of Geneva, Geneva, Switzerland; 2 Department of Zoology, University of Oxford, Oxford, United Kingdom; University of Toronto, Canada

## Abstract

With the rising development of bacterial resistance the search for new medical treatments beyond conventional antimicrobials has become a key aim of public health research. Possible innovative strategies include the inhibition of bacterial virulence. However, consideration must be given to the evolutionary and environmental consequences of such new interventions. Virulence and cooperative social behaviour of the bacterium *Pseudomonas aeruginosa* rely on the quorum-sensing (QS) controlled production of extracellular products (public goods). Hence QS is an attractive target for anti-virulence interventions. During colonization, non-cooperating (and hence less virulent) *P. aeruginosa* QS-mutants, benefiting from public goods provided by wild type isolates, naturally increase in frequency providing a relative protection from invasive infection. We hypothesized that inhibition of QS-mediated gene expression removes this growth advantage and selection of less virulent QS-mutants, and maintains the predominance of more virulent QS-wild type bacteria. We addressed this possibility in a placebo-controlled trial investigating the anti-QS properties of azithromycin, a macrolide antibiotic devoid of bactericidal activity on *P. aeruginosa*, but interfering with QS, in intubated patients colonized by *P. aeruginosa*. In the absence of azithromycin, non-cooperating (and hence less virulent) *lasR* (QS)-mutants increased in frequency over time. Azithromycin significantly reduced QS-gene expression measured directly in tracheal aspirates. Concomitantly the advantage of *lasR*-mutants was lost and virulent wild-type isolates predominated during azithromycin treatment. We confirmed these results *in vitro* with fitness and invasion experiments. Azithromycin reduced growth rate of the wild-type, but not of the *lasR*-mutant. Furthermore, the *lasR*-mutant efficiently invaded wild-type populations in the absence, but not in the presence of azithromycin. These *in vivo* and *in vitro* results demonstrate that anti-virulence interventions based on QS-blockade diminish natural selection towards reduced virulence and therefore may increase the prevalence of more virulent genotypes in the Hospital environment. More generally, the impact of intervention on the evolution of virulence of pathogenic bacteria should be assessed.

***Trial Registration:*** ClinicalTrials.gov NCT00610623

## Author Summary

With the rising development of antibiotic resistance and rapid spread of nosocomial pathogens, the search for new treatments beyond conventional antibiotics becomes a key aim of public health research. As such, anti-virulence therapies might be alternative antimicrobial strategies. However, consideration must be given to the potential evolutionary and environmental consequences of such interventions. Here we demonstrate a significant evolutionary impact of an anti-virulence intervention. Virulence and cooperative social behaviour of *Pseudomonas aeruginosa* rely on the quorum-sensing (QS) controlled production of extracellular products. In the absence of a specific intervention, non-cooperating (and hence less virulent) QS-mutants exploit and benefit from products provided by wild type isolates. As a consequence these less virulent mutants increase in frequency and provide a relative protection against infection to a colonized patient. In contrast, when QS-gene expression is reduced by the QS-inhibiting drug azithromycin, this advantage of QS-mutants is lost and virulent isolates predominate both in colonized patients and during *in vitro* experiments. These results suggest that QS-blockade may increase the prevalence of more virulent QS-responders among colonizing isolates in the hospital environment. More generally, the impact of anti-virulence interventions on the ecology and evolution of virulence of pathogenic bacteria needs to be assessed.

## Introduction

Anti-virulence therapies have been recently suggested as alternative strategies to circumvent the growing problem of antibiotic resistance [1], [2]. In *P. aeruginosa* inhibition of Quorum-Sensing (QS) seems particularly attractive as QS regulates many virulence determinants of this pathogen [3]. Azithromycin is a widely used macrolide antibiotic without significant bactericidal activity on *P. aeruginosa* [4]. Recent studies suggest azithromycin might be of benefit against this bacterium because it interferes with the QS-circuit and thereby inhibits the expression of a wide range of extracellular virulence factors [5]. Inhibition of QS is likely to have important evolutionary consequences for *P. aeruginosa*. Both *in vitro* and *in vivo* studies suggest that mutants (QS-cheats) that don't respond to QS (specifically, mutants that are defective in one of the QS-receptors, LasR) can have a selective advantage in the presence of QS-wildtypes [6], [7]. This has been recently demonstrated during colonization of untreated colonized patients in whom QS-cheats accumulated over time [8]. The most likely explanation for this advantage is that the mutants exploit the wild type public goods, without paying the metabolic cost of their production [9]–[11]; although other direct costs of QS in clinical contexts can't be ruled out [12]–[14]. Regardless of the reasons why QS-mutants have a fitness advantage, this advantage is unlikely to be realised if QS is blocked in wild type bacteria. Azithromycin (or any QS-blocker) will therefore reduce, or remove, selection for less virulent QS-cheats and maintain the predominance of more virulent QS-wild type bacteria.

We tested this hypothesis by following the evolutionary dynamics of QS (*lasR*) mutants in intubated patients colonised by *P. aeruginosa*, during a placebo controlled clinical trial evaluating the prevention of pneumonia by azithromycin. Whereas the proportion of *lasR* mutants rapidly increased in the untreated control patients, the proportion did not change in the azithromycin-treated patients. This fitness advantage in the absence, but not the presence, of azithromycin was similarly observed *in vitro*. More generally, the impact of intervention on the evolution of virulence of pathogenic bacteria should be assessed [15].

## Results/Discussion

**Figure 1:**
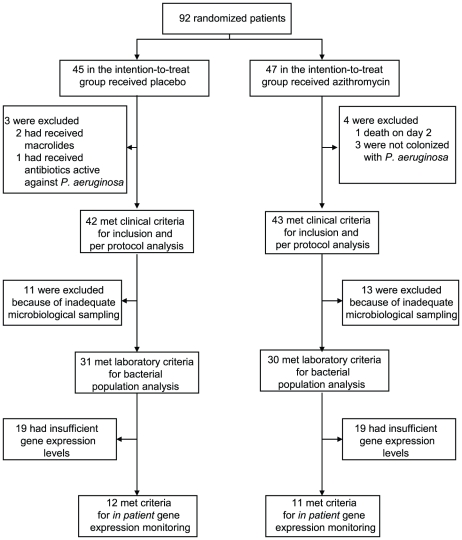
Patient enrollment and follow-up.

We tested the hypothesis that azithromycin reduces selection for QS-cheats by following prospectively 92 intubated patients (colonization times of three to twenty days), colonized by *P. aeruginosa* and hospitalized in intensive care units of seventeen European Hospitals, participating in a placebo controlled azithromycin pneumonia prevention trial (*see material and methods*). Importantly, antibiotic treatments active against *P. aeruginosa* were forbidden during the trial. We collected a single *P. aeruginosa* isolate per patient per day from tracheal aspirates and estimated total density of *P. aeruginosa* bacteria in the aspirates through genomic copy numbers. Adequate microbiological sampling for bacterial population analysis was available for 61 patients (31 placebo and 30 azithromycin) of the initial 92 randomized patients in the intention-to-treat protocol (Figure 1).

### QS-inhibition *in patient*

**Figure 2:**
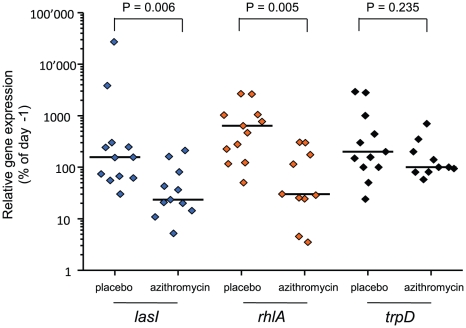
*In patient* QS-inhibition in azithromycin-treated patients. *In patient* QS-gene expression was determined as described. Tracheal aspirates from both day −1 and day x with bacterial RNA of adequate quality were available for twelve placebo and eleven azithromycin patients. Expression of QS-circuit gene *lasI*, QS-target gene *rhlA* and QS-independent gene *trpD* measured in tracheal aspirates is shown as the relative value (%) of the last accessible day (Dx) compared to day −1 (set as 100%). A horizontal line indicates the median expression levels. P values were calculated using Mann-Whitney tests.

We monitored QS-gene expression directly in tracheal aspirates to document the “*in patient*” QS-inhibition by azithromycin. Azithromycin significantly reduced the expression of both QS-circuit (*lasI*; Mann-Whitney test, *P* = 0.006) as well as QS-target (*rhlA*; *P* = 0.005) genes, whereas it did not affect expression of the QS-independent gene *trpD* (*P*>0.2) (Figure 2). It is of course possible that azithromycin inhibited the expression of some other genes unrelated to QS. However microarray data have shown that QS-regulated genes were among those whose expression was most severely affected by azithromycin [16].

**Figure 3:**
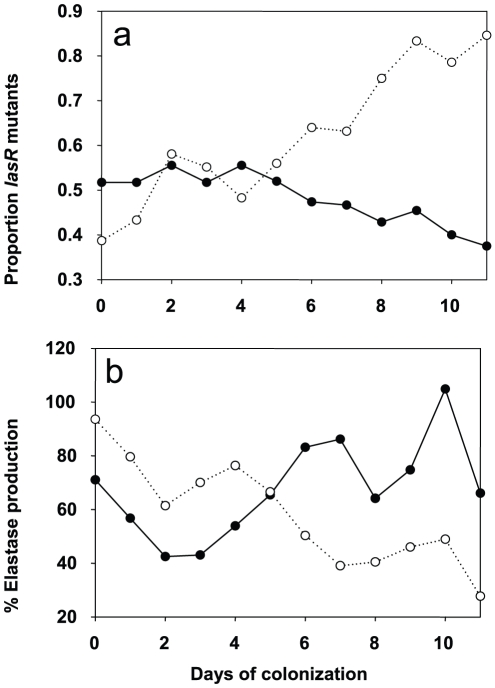
Evolution of *lasR* mutants and elastase production in azithromycin-treated and untreated patients. Change in the proportion of *lasR* mutants (a) and mean elastase production (b) through time. Solid lines and closed symbols indicate azithromycin-treated patients, and dashed lines and open symbols indicate placebo group. Note that data is presented to day 11 of colonization, despite some samples being collected up to 20 days, because of very small sample sizes (six isolates) by day 12 in the azithromycin-treated group. However, qualitatively identical results were obtained when the whole data set was analysed. The change in the proportion of *lasR* mutants and elastase through time was analysed using logistic regression, corrected for under-dispersion, and General linear Modelling, respectively in GenStat 10.

We determined the evolution of *P. aeruginosa* QS in patients by first measuring the production of elastase, which is under the control of the *lasR* QS-system [17] from the 650 collected isolates (data not shown). Variations in elastase activity correlated with mutations in *lasR* between independent wild type and mutant *lasR* alleles (Mann-Whitney: *P*<0.001), as determined by sequencing this gene in the first isolate obtained from each patient, and then in subsequent isolates presenting a different QS-phenotype (Figure 3). Mutations in *lasR* were therefore reducing the expression of elastase, and by inference, other *lasR*-regulated genes. Whereas the proportion of *lasR* mutants significantly increased through time in the 31 control group patients (Figure 3a; logistic regression: *F*_1,10_ = 65.36, *P*<0.001), there was a small, decline in the proportion of *lasR* mutants in the 30 azithromycin treated patients (Figure 3a; *F*_1,10_ = 32.58, *P*<0.001; test of whether slopes differ (treatment by time interaction) in full model: *F*_1, 20_ = 77.6; *P*<0.001). In agreement with this observation, isolates from placebo patients showed decreasing mean elastase levels *in vitro* (Figure 3b; *F*_1,10_ = 41.12, *P*<0.001), while isolates from the azithromycin treated group showed a concomitant increase through time (Figure 3b; *F*_1,10_ = 41.12, *P*<0.001; treatment by time interaction in full model: *F*_1,20_ = 26.46, *P*<0.001).

These data are consistent with the hypothesis that azithromycin treatment removes any advantage of QS-mutants, because QS is blocked in the wildtype population. There are, however, a number of alternative explanations, particularly as bacterial densities, based on mean *P. aeruginosa* genomic copy numbers, were twice as large in the placebo compared to the azithromycin group (7×10^6^ and 1.2×10^7^; *t* = 1.96, *P* = 0.06). First, azithromycin-imposed reduction in densities could reflect reductions in growth rate, and this could simply have slowed down the rate at which *lasR* mutants change in frequency. We can however rule this out as a primary explanation for our data, because azithromycin did not only cause a quantitative change in the frequency of *lasR* mutants, but also a qualitative change: *lasR* mutants decreased in frequency during azithromycin treatment, whereas they increased in the placebo group (Figure 3). Second, it is possible that azithromycin may reduce selection for *lasR* mutants if reductions in QS-mediated public goods production results from reductions in bacterial density caused by azithromycin. Third, azithromycin may directly inhibit the growth of *lasR* mutants more than wildtype bacteria, explaining why there was a small reduction in the frequency of *lasR* mutants following azithromycin treatment. We address these possibilities below. Furthermore we cannot exclude that azithromycin influenced the structure of the resident bacterial flora of the patients which could in turn influence the *P. aeruginosa* population and its virulence properties [18].

### QS-inhibition *in vitro*

To aid the interpretation of the clinical data, we carried out *in vitro* experiments with wild type *P. aeruginosa* (PAO1) and an isogenic *lasR* mutant in the presence and absence of azithromycin. Danesi et al. [19] measured azithromycin concentrations of 9 mg/kg in lung tissue of patients receiving a comparable dosing regimen as those in our study, hence we used similar concentrations (5 and 10 mg/l) for our *in vitro* experiments. We first measured growth rates in media where the primary nutrient source is protein (BSA), making *lasR*-controlled expression of proteases necessary for the production of useable amino acids [6].

**Figure 4:**
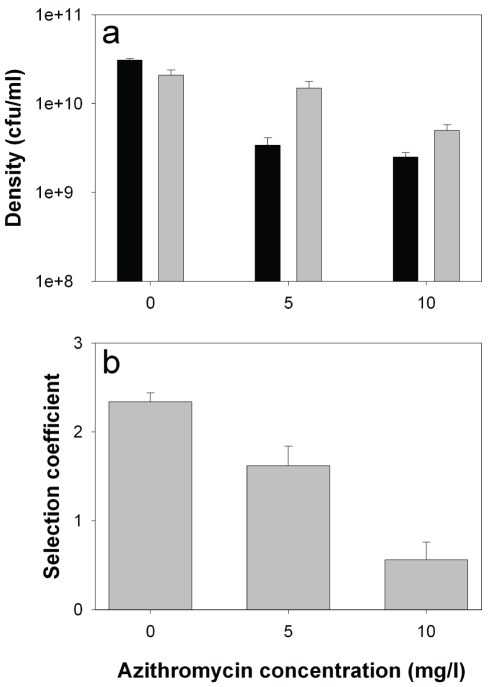
*lasR* mutant growth rates and invasion of wild type populations in the presence and absence of azithromycin. *In vitro* densities of wildtype (black) and *lasR* mutant (grey) after 72 hours growth in M9 salts BSA medium (a), and selection coefficients of *lasR* mutant relative to wildtype (b) as a function of azithromycin (AZM). Bars show means (± SEM) of six replicates. All differences (wildtype versus *lasR*) in the presence of azithromycin are statistically significant (p<0.05).

Consistent with previous studies [6], growth rate of the *lasR* mutant in monoculture was reduced relative to the wildtype (by approximately 50%) in the absence of azithromycin, demonstrating an advantage of QS in this environment (Figure 4a; 2-sample *t*-test: *P*<0.05). The addition of azithromycin reduced densities of both genotypes (linear effect of azithromycin: *F*_1,29_ = 71.2, *P*<0.001), but this reduction was much greater for the wildtype than the *lasR* mutant (interaction between concentration and genotype: *F*_1,29_ = 6.92, *P* = 0.013), confirming a role of azithromycin in suppressing the production of QS-controlled exoproducts. Given that azithromycin inhibits wildtype growth more than that of the *lasR* mutant, we are unable to explain the slight drop in the frequency of *lasR* mutants in the patients.

We next measured the fitness of the *lasR* mutant invading wildtype populations (1∶100 ratio). Consistent with the *in vivo* results, we found that the fitness advantage of *lasR* mutants was decreased with increasing azithromycin concentration (Figure 4b; Linear effect of azithromycin concentration of fitness of *lasR* mutant: *F*_1,16_ = 48.41, *P*<0.001). Unlike in the clinical context, the *lasR* mutant still had a slight fitness advantage over the wildtype at the highest concentration of azithromycin used (10 mg/l), suggesting, unsurprisingly, that additional variables influence the fitness of *lasR* mutants *in vivo*. These results strongly suggest that the major advantage of *lasR* mutants *in vitro* (and presumably *in vivo*) is their ability to exploit wildtype public goods [8], and that azithromycin removes this advantage because less public good (elastase) is produced. However, the data do not distinguish between azithromycin directly inhibiting elastase production, or indirectly through density reductions, or both.

### Conclusions

We have shown that azithromycin treatment can prevent selection for *lasR* mutants, and consequently increase the proportion of wild type *P. aeruginosa* in colonized patients. A number of not mutually exclusive factors may help to explain this pattern, but the data suggests that the primary reason is because azithromycin blocks QS. Blocking QS prevents *lasR* mutants from exploiting the public goods provided by the wildtype, and reduces any direct costs associated with QS, such as production of extracellular products that are not of benefit in this particular environment [8]. Both *in vitro* and *in patient* data obtained from the clinical trial suggest a key role of QS-dependent virulence for the development of infection (Köhler et al., submitted), and support the general consensus from studies using animal models showing that QS-expression (and public goods production in general) is associated with increased virulence [2], [6], [20]–[23]. Azithromycin is therefore likely to be of clinical benefit to a treated patient by inhibiting the QS-dependent virulence during the course of the treatment. However, when treatment is discontinued the patient is at risk of being colonized by highly virulent bacteria, with the potential of late onset infections. Moreover a wider use of such anti-virulence interventions may also increase the prevalence of highly virulent QS-wild type isolates within the hospital. More generally, any intervention that reduces bacterial densities is also likely to result in a reduced selective advantage of less virulent mutants that do not make public goods [24]. The study highlights the need to carefully consider both the short and longer term implications of anti-virulence therapy and other interventions (such as vaccines [15]) on pathogen virulence.

## Materials and Methods

### Ethic statement

We obtained approval for this study by the “Commission Centrale d'Ethique de la Recherche sur l'Etre Humain des Hôpitaux Universitaire de Genève”. Written informed consent from all patients or their legal representatives was obtained according to legal and ethical considerations.

### Patients and clinical collection

This randomized, placebo-controlled, double blind study (ANB 006#2001, ClinicalTrials.gov ID#NCT00610623) was designed to assess the efficacy of azithromycin as a quorum-sensing inhibitor in preventing the occurrence of *P. aeruginosa* pneumonia in ventilated patients with documented colonization. Twenty-one European centers participated in this trial; eight in France, four in Spain, three in Belgium, three in Poland, two in Serbia and one in Switzerland. We screened mechanically-ventilated patients for respiratory tract colonization by *P. aeruginosa* every 48 hours. Neutropenic patients and patients treated with immunosuppressive drugs were not eligible. Patients with ongoing *P. aeruginosa* infection, having received macrolides or antibiotics active against the colonizing *P. aeruginosa* isolate during the last 14 days were excluded. Patients with proven colonization by *P. aeruginosa* were randomized (D-1) and received either placebo or 300 mg per day iv azithromycin in a double blind fashion for a maximum of 20 days (D1 to Dx). During the study, only the administration of antibiotics inactive against *P. aeruginosa* was allowed. Detailed information on study design is available in supporting information Protocol S1 and Checklist S1. Patient characteristics and clinical outcome of the study are published elsewhere (van Delden et al., submitted). Starting the first day of proven colonization (D-1), we collected tracheal aspirates (0.3 to 5 ml) and one *P. aeruginoa* isolate (collection period: 3–20 days) at 24 hours intervals. Samples were frozen at −80°C on site within 15 minutes, and sent on dry ice to the reference research laboratory at the University Hospital Geneva, where all analyses were performed in a blind fashion. The logit-transformed proportion of patients whose isolate was a *lasR* mutant was analyzed by logistic regression, with time (a covariate), treatment (placebo or azithromycin) and the interaction fitted in GenStat v10. Data were over dispersed, so a scaling factor to equalize the residual error and degrees of freedom was employed.

### *In patient* gene expression analysis

From prospectively collected tracheal aspirates we extracted total genomic DNA and total RNA (for details see supporting information Text S1). We detected (>10^4^ genomic copies/g aspirate) *P. aeruginosa* DNA in 98% of the aspirates, confirming the colonization by this organism. In the RNA extractions, we detected expression (>5×10^4^ copies/g aspirate) of the *rpsL* housekeeping gene in 80% of the aspirates. This indicates that quality of both sample handling and RNA extractions were sufficient to detect bacterial gene expression in the majority of the tracheal aspirates. As a second control for the quality of the RNA extracts from clinical samples we plotted the amount of *P. aeruginosa* bacteria as determined by qRT-PCR from the genomic DNA extractions against the expression of the *rpsL* housekeeping gene. We observed a good correlation between these two variables (*r* = 0.69, P<0.001).

### Determination of bacterial loads

The number of *P. aeruginosa* in aspirates was determined by qRT-PCR of genomic DNA preparations. Aliquots of genomic DNA preparations were diluted 10 fold into H_2_O and 3 µl of this dilution were added to the PCR reaction mix containing 1× Quantitect Sybr Green Master Mix and 600 nM primers in a total volume of 15 µl. PCR conditions were as described below for cDNA analysis. A standard curve was obtained by addition of 10-fold dilutions of a *P. aeruginosa* culture to an aspirate collected from a patient not colonized by this organism. Genomic DNA was then isolated as described above and quantified by qRT-PCR. Under these conditions, we detected 10^4^ CFU/g aspirate. Standard curves yielded reproducible values during the 3-month analysis period. *P. aeruginosa* was found in the aspirates at levels varying from 4×10^4^–1.8×10^8^ CFU/g.

### *In vitro* experiments

*P. aeruginosa* strain PAO1 was competed against a rare invading isogenic *lasR* knockout mutant [25] in 200 µl M9 minimal salts medium supplemented with 1% BSA [6] in 96-well plates, shaken at 200 rpm at 37°C in the presence or absence of azithromycin (5 and 10 mg/l) for 72 hours. Six wells per environment were inoculated with 10^7^ cells of overnight cultures (grown in LB medium at 37°C), at a ratio of 1∶100 *lasR* mutant: wild type. Selection coefficients of the *lasR* mutants was calculated as the differences in malthusian parameters (ln(final density/starting density) as previously described [26], with cell counts determined by plating on LB agar and LB supplemented with 50 mg/l tetracycline. A selection coefficient of zero indicates that strains have equal fitness. Selection coefficients were regressed against azithromycin concentration. Densities (colony forming units) of pure cultures (6 replicates per treatment) under the same conditions were determined at the same time. Densities were log_10_-transformed, to meet assumption of general linear models, and concentration (a covariate), strain and the interaction fitted in GenStat.

## Supporting Information

Text S1 Supplementary material and methods
(0.03 MB DOC)

Protocol S1 Trial protocol
(0.36 MB PDF)

Checklist S1 CONSORT Checklist
(0.06 MB DOC)
