# Supplementary material for: Quorum Sensing Inhibition Selects for Virulence and Cooperation in Pseudomonas aeruginosa
Source: PLoS Pathog. 2010 May 6;6(5):e1000883. doi: 10.1371/journal.ppat.1000883 (PMC2865528; doi:10.1371/journal.ppat.1000883)
Supplement: Text S1 — Supplementary material and methods (0.03 MB DOC) [file ppat.1000883.s001.doc]

**Extraction of RNA and DNA from aspirates.** Two ml RNA protect bacteria solution and dithiothreitol (0.64 mM final concentration) were added to each g of aspirate. After vortexing and subsequent shaking at 37 °C for 10 min, 0.5 ml aliquots of this homogenate were used for RNA and autoinducer extraction, and 50 μl aliquots for genomic DNA extraction. All extractions were done in duplicate. Samples for RNA extraction were centrifuged and the pellet resuspended in 1 ml trizol reagent. Chloroform (0.2 ml) was added and the suspension vigorously shaken. After 3 min at RT, samples were centrifuged and the aqueous phase precipitated with 0.5 ml isopropanol. After centrifugation, the RNA pellet was washed with 1 ml 70% ethanol. Once dried, the RNA pellet was resuspended in 43 μl RNAse-free H_2_O by heating for 10 min at 56 °C. To remove residual DNA, a mix containing the RNA preparation, 1 x DNAse buffer, 1 unit RNAse inhibitor and 5 units RNAse-free DNAse were mixed and incubated for 40 min at 37 °C. The RNA was then purified using RNeasy columns, according to the instructions of the supplier.

Genomic DNA was extracted using the DNAzol solution. The final DNA pellet was dissolved in 0.2 ml 8 mM NaOH by heating at 56 °C for 10 min. RNA and DNA were quantified in a spectrophotometer. The primer pair rpsL-F (5’ GCAAGCGCATGGTCGACAAGA-3’ and rpsL-R (CGCTGTGCTCTTGCAGGTTGTGA-3’) was tested for specificity against genomic DNA from clinical isolates of *Streptococcus pneumoniae*, *Klebsiella* *pneumoniae*, *Escherichia coli* and *Acinetobacter baumannii*. An amplification signal was obtained only with genomic DNA isolated from *P. aeruginosa*.

**Preparation and quantification of cDNA**. To determine “*in patient*” bacterial gene expression, we extracted total RNA from tracheal aspirates and quantified bacterial gene expression by qRT-PCR. Total RNA (500 ng) was incubated at 60 °C for 5 min with 20 ng random hexamer primers and 0.8 mM each dNTPs, before chilling the mix on ice. To this mix, 80 units of RNAsin, 1 x first strand buffer and MgCl_2_ (2.5 mM final concentration) were added up to 38 ml. The mix was left at RT for 10 min and then 2 min at 42 °C. At this point, 1 ml (1 unit) of reverse transcriptase was added to a 19 ml aliquot of the mix. The remainder of the mix served as negative control. Reverse transcription proceeded for 50 min at 42 °C. After inactivation of the enzyme at 70 °C for 15 min, 60 ml RNAse-free H_2_O were added to obtain a final volume of 80 ml. Primers used for amplification were designed based on the PAO1 genome sequence, using the Primer3 software (<http://frodo.wi.mit.edu/cgi-bin/primer3/primer3.cgi/primer3_www.cgi>), and verified for unique hybridization on the PAO1 genome sequence ([www.v2.pseudomonas.com](http://www.v2.pseudomonas.com/)). Each PCR reaction contained 1 x Quantitect SybrGreen Master Mix, 600 nM each primer and 3 ml of a 4-fold dilution of template cDNA (or RT- controls) in a total volume of 15 ml. The cycling parameters used were as follows: 15 min at 95 °C, 40 amplification cycles of 95 °C for 20 sec, 60 °C for 20 sec and 72 °C for 30 sec. Acquisition was at 72 °C. Melting curves done at the end of the run showed a single product peak. Data were analyzed with the RotorGene software (version 6.0). The amount of cDNA was deduced by comparison to standard curves which were obtained by serial dilutions of plasmid encoded copies of the corresponding genes. Amplification of samples that had not been reverse transcribed, indicated incomplete digestion of genomic DNA. Therefore, RT- values were subtracted for each sample, and final results were expressed as the difference of (copies cDNA – copies RT-) / g aspirate, hereafter referred to as copies / g aspirate. RNA and DNA preparations were done in duplicate for each aspirate, and results given as mean of two determinations. The linearity and specificity of our assay were tested by adding serial dilutions of a *P. aeruginosa* laboratory strain culture to a tracheal aspirate that contained Gram-positive and Gram-negative bacteria but no *P. aeruginosa*. Genomic and total RNA were isolated and their respective amounts determined by comparison with standard curves. A linear correlation over at least three orders of magnitude was observed between the amount of bacteria calculated from the genomic DNA and the amount of *rpsL* or *lasI* cDNA (data not shown). As negative control, we also added dilutions of a *lasI* mutant strain. As expected, *rpsL* but not no *lasI* cDNA was detected (> 1 × 10^4^ copies / g aspirate) in these samples (data not shown).
