# Supplementary material for: Quorum Sensing Inhibition Selects for Virulence and Cooperation in Pseudomonas aeruginosa
Source: PLoS Pathog. 2010 May 6;6(5):e1000883. doi: 10.1371/journal.ppat.1000883 (PMC2865528; doi:10.1371/journal.ppat.1000883)
Supplement: Protocol S1 — Trial protocol (0.36 MB PDF) [file ppat.1000883.s002.pdf]

Anbics Management-Services AG

CLINICAL STUDY PROTOCOL

Azithromycin

ANB 006 #2001

PROOF-OF CONCEPT STUDY TO INVESTIGATE THE IMPACT OF AZITHROMYCIN ADMINISTERED I.V. VS. PLACEBO ON THE PREVENTION OF PNEUMONIA IN VENTILATED PATIENTS COLONIZED WITH *PSEUDOMONAS AERUGINOSA*

Multinational study

Coordinating Investigator
Dr. Jean Carlet
Chef de Service
Service de Réanimation Polyvalente
Fondation Hôpital Saint-Joseph
185, Rue Raymond Losserand
F-75014 Paris
France

Clinical Pharmacologist
Dr. Françoise Brunner
Brunner Naga
Health Science Consulting
Im Stock 14
CH-8330 Pfaeffikon-Zurich
Tel ++41-(0)1-995 1568
Fax ++41-(0)1-995 1569

Centre *xx*
Principal Investigator
Department
Hospital
Address
City, mail code
Country
Tel
Fax

Quintiles Project Director
Philip Roopram
Quintiles Limited
Station House
Market Street
Bracknell RG12 1HX
United Kingdom
Tel: ++44-(0)1344 708 767
Fax: ++44-(0) 1344 708 777

Sponsor
Anbics Management-Services AG
Technoparkstrasse 1
CH-8005 Zurich
Tel: ++41-(0)1-445 3180
Fax: ++41-(0)1-445 3919

Site Monitor
See separate Centre & Monitor List

This protocol is the confidential information of Anbics Management-Services AG (abbreviated Anbics AG) and is intended solely for the guidance of the clinical investigation. This protocol may not be disclosed to parties not associated with the clinical investigation or used for any purpose without the prior written consent of Anbics AG

CONFIDENTIAL

TABLE OF CONTENTS

PROTOCOL SYNOPSIS **6**

STUDY SCHEDULE **10**

ABBREVIATIONS AND DEFINITIONS **11**

1. INTRODUCTION AND RATIONALE FOR STUDY DESIGN **13**

1.1 Introduction **13**

1.2 Quorum-Sensing **13**

2. STUDY OBJECTIVES **18**

3. STUDY DESIGN **19**

4. SELECTION OF SUBJECTS **20**

4.1 Number of Subjects **20**

4.2 Inclusion Criteria **20**

4.3 Exclusion Criteria **20**

5. STUDY TREATMENTS **22**

5.1 Details of Study Treatments **22**

5.2 Dosage Schedule **22**

5.3 Treatment Assignment **23**

5.4 Blinding, Packaging, and Labeling **23**

5.5 Supplies and Accountability **24**

5.6 Compliance **24**

6. PRIOR AND CONCOMITANT ILLNESSES AND TREATMENTS **25**

6.1 Prior and Concomitant Illnesses **25**

6.2 Prior and Concomitant Treatments - Antibiotherapy **25**

7. STUDY PROCEDURES AND SCHEDULE **27**

7.1 Overview of Data Collection **27**

7.1.1. Clinical efficacy data (collected at each clinical site) 27

7.1.2. Pharmacodynamic data performed in local laboratory 27

7.1.3 Pharmacodynamic data performed in Geneva laboratory 28

7.1.4. Safety data 28

7.2 Description of Study Days **28**
7.2.1 General Recommendations and Procedures for Study 28
7.2.2 Prestudy Screening (Day -1) 29
7.2.3 Study days 30
7.2.4. Day x (Day of pneumonia) 33
7.2.5 Day n-1 (last day of treatment) 34
7.2.6 Poststudy (Day n + 7) 35
7.3. Methods of evaluation **35**

8. ADVERSE EVENTS 37
8.1 Definitions **37**
8.1.1 Adverse Event 37
8.1.2 Serious Adverse Event 37
8.2 Period of Observation **38**
8.3 Documentation and Reporting of Adverse Events by Investigator **38**

9. WITHDRAWALS 40
9.1 Withdrawal of Subjects **40**
9.2 Replacement of Subjects **40**

10. EMERGENCY PROCEDURES 41
10.1 Emergency Sponsor Contact **41**
10.2 Emergency Identification of Study Medication **41**
10.3 Emergency Treatment **41**

11. STATISTICAL PROCEDURES 42
11.1 Analysis Variables **42**
11.2 Study Populations **42**
11.3 Statistical Methods **42**
11.4 Interim Analysis **43**
11.5 Sample Size Justification **43**

12. ETHICAL AND LEGAL ASPECTS 45
12.1 Good Clinical Practice **45**
12.2 Delegation of Investigator Responsibilities **45**
12.3 Subject Information and Informed Consent **45**
12.4 Confidentiality **46**
12.5 Protocol Amendments **46**
12.6 Approval of the Study Protocol and Amendments **46**

12.7 Ongoing Information for the Ethical Review Committee (ERC)....46
12.8 Premature Closure of the Study....46
12.9 Record Retention....47
12.10 Liability and Insurance....47

13. STUDY MONITORING AND AUDITING....48
13.1 Study Monitoring....48
13.2 Source Data Verification and On-Site Audits....48

14. DOCUMENTATION AND USE OF STUDY FINDINGS....49
14.1 Documentation of Study Findings....49
14.2 Use of Study Findings....49

15. STUDY DURATION AND DATES....50

16. DECLARATIONS OF SPONSOR AND INVESTIGATOR....51
16.1 Declaration of Sponsor....51
16.2 Declaration of Investigator....52

17. REFERENCES....54

APPENDIX A....57
Child Pugh's Score....57

APPENDIX B....58
Laboratory Safety....58

APPENDIX C....59
Preparation of Drug Treatment Solutions for i.v. Administration....59

APPENDIX D....60
Collection and Storage of Tracheal Aspirates....60

APPENDIX E....62
Collection and Assessment of Bronchial Alveolar Lavage (BAL),....62
Bronchiotracheal Aspirate or Protected Brush Specimen (PBS) on Day x....62

APPENDIX F....64
Recovery and Storage of Endotracheal Device....64

APPENDIX G....65
Transport of Biological Specimens & Endotracheal Device....65

APPENDIX H **66**
Acute Physiology Chronic Health Evaluation (APACHE II) **66**

APPENDIX I **68**
Clinical Pulmonary Infection Score (CPIS) **68**

APPENDIX J **69**
Electrocardiogram (ECG) **69**

PROTOCOL SYNOPSIS

Azithromycin

ANB 006 #2001

Title

PROOF-OF CONCEPT STUDY TO INVESTIGATE THE IMPACT OF AZITHROMYCIN ADMINISTERED I.V. VS. PLACEBO ON THE PREVENTION OF PNEUMONIA IN VENTILATED PATIENTS COLONIZED WITH *PSEUDOMONAS AERUGINOSA*

Coordinating Investigator

Dr. Jean Carlet, Paris

<u>Centre *xx*</u>
Principal Investigator
Department
Hospital
Address
City, mail code
Country

List of Centres by Country

Each country centre list will include:

- National Coordinating Investigator
- Each site with:
    - Site number
    - Principal Investigator
    - Subinvestigators
    - Address
- Quintiles Study Monitor(s)

Phase IIa

Multinational and multicentric

Indication

Prevention of pneumonia in ventilated patients colonized with *Pseudomonas aeruginosa*

Objectives

Primary objective

- To assess the clinical efficacy of azithromycin 300 mg i.v., used as a quorum-sensing blocker, compared to placebo for preventing or delaying the occurrence of pneumonia in ventilated patients colonized with *Pseudomonas aeruginosa*

Secondary objectives

- To assess other clinical efficacy variables
- To compare safety profile in target population
- To demonstrate using both *in vivo* and *in vitro* parameters whether azithromycin can be used clinically as a quorum-sensing blocker against *P. aeruginosa*
- To determine whether *P. aeruginosa* can develop resistance to the quorum-sensing blocker effect of azithromycin

- To check whether prolonged treatment with azithromycin may induce resistance to any of 11 antipseudomonal antibiotics (list in Section 6.2.)
- To verify whether emergence of resistance to other bacteriae may be elicited by assessing rate of infections in both study groups (azithromycin vs. placebo)

Design

2-parallel groups, double blind, randomized, placebo controlled, multiple i.v. dose administered once daily

Population

- Intubated patients
- Age 18 to 75 years
- Males and non pregnant females
- Respiratory tract colonized by *Pseudomonas aeruginosa* as documented by tracheal aspirate

Sample size

- Recruit a total of 120 to 240 patients in order to achieve 100 to 200 assessable cases
- Stop recruitment when 10 *P. aeruginosa* pneumonia cases have been documented and confirmed by panel of experts under blinded conditions

Treatments

- 300 mg azithromycin as i.v. solution at 2 mg/mL or saline (placebo), infused daily over 1 hour, until extubation (Day n-1), occurrence of pneumonia (Day x), exitus, or for a maximum of 20 cconsecutive days (Day 20)

Clinical efficacy (clinical site)

- Occurence of *P. aeruginosa* pneumonia (primary variable)

  The diagnosis of pneumonia will be assessed by an independent panel of experts under blinded conditions prior to stopping the study
- Occurrence of death (excluded if death within 3 days and not elicited by *P. aeruginosa*)
- Time to pneumonia (Day x), to exitus, or to bacteremia
- Time to extubation
- Overall outcome (until Day n+7)
- Duration of hospital and ICU stay
- Cost assessment
- Pa_{O2} / FI_{O2}
- Occurrence of infections to other bacterial strain

Pharmacodynamic data

Tracheal aspirates (local laboratory, on Aspirate 1)

- Collection daily

- Quantitative culture for *P.aeruginosa* **(**CFU/mL) on Day -1, 1, 3, 6, 9, 12, 15, 18, and last day (i.e. Day x, or Day n-1, or Day 20)
- Isolate *P. aeruginosa* strain daily and store at –70°C on skim milk and glycerol
- Emergence of resistance (MIC to *P.aeruginosa* in tracheal aspirate for 11 standard antipseudomonal antibiotics on Day -1, and on last day (Day x, or Day 20, or Day n-1, or Day 20, see list Section 6.2)
- CFU for other strains from tracheal aspirates if indicated

BAL on Day x (or protected brush specimen (PBS), or aspirate local laboratory)

- Only for patients with suspected pneumonia to confirm diagnosis
- CFU/mL
- Cellularity

Tracheal aspirates (*in situ*, Geneva laboratory on Aspirate 2)

- Collection daily at study site, storage at –70°C prior to transfer to Geneva
  - Day –1, 1, 3, 6, 9,…, last day (Day x, or Day n-1, or Day 20) for all patients
  - Daily for pneumonia (n=10) and control patients (n=20), selected on a site basis
  - Daily for patients under antibiotherapy
- Parameters
  - Content for autoinducer homoserine lactones: 3-oxo-C_{12}-HSL and C_4-HSL **and/or**
  - mRNA for LasB elastase (lasI, rhlI)

Respiration device on Day n-1 (Geneva laboratory)

- CFU/cm^2
- Autoinducer content

Isolated *P.aeruginosa* strains from tracheal aspirate 1 and respiration device (Day n-1) (*in vitro*, Geneva laboratory)

- Day –1, 1, 3, 6, 9,…, last day (Day x, or Day n-1, or Day 20) for all patients
- Daily for pneumonia (n=10) and selected control patients (n=20)
- Daily for patients under antibiotherapy
- *In vitro* virulence factor production (elastase, protease, rhamnolipids, cytotoxicity)
- *In vitro* autoinducer production (3-oxo-C_{12}-HSL and C_4-HSL)
- Azithromycin gradient plates for detection of rhamnolipid production (Day -1, Day x or Day n-1 or Day 20) to detect possible resistance mechanisms

Safety data

- Adverse event profile (reported by subject, observed by investigator)
- Local tolerance (only if peripheral infusion site)
- Standard laboratory tests (see Appendix B)
- Blood gases
- Vital signs (blood pressure, heart rate, body temperature)

- 12-lead electrocardiogram (ECG)
- Emergence of non Pseudomonas infections

Statistical procedures

The primary analysis variable will be the occurrence of pneumonia, using Fischer's exact test. Other clinical and pharmacodynamic variables measured *in vivo* and *in vitro* will be analyzed by Fischer's test or chi-square for categorical variables (presence or absence of condition) whereas continuous variables will be compared using the t-test or the Mann-Whitney test, as considered best indicated. Appropriate graphs for key parameters as well as tables for all analyzed variables will be provided.

Descriptive analysis and listings will be produced for all safety variables (laboratory, adverse events, local tolerance).

Study duration and dates

The duration of this study is expected to be approximately 1 year, depending however heavily on number of assessable patients required (100 to 200) to achieve 10 pneumonia cases elicited by *P. aeruginosa*. Subject recruitment proposed is to start in November 2002 with study completion anticipated in Autumn 2003 to include a maximum of 240 cases for achieving 200 assessable patients.

STUDY SCHEDULE

ABBREVIATIONS AND DEFINITIONS

A-aD0$_2$	Alveolar-arterial P_{O2} difference (gradient)
aPTT	Activated partial thromboplastin time
ADR	Adverse drug reaction
AE	Adverse event
AIDS	Acquired immunodeficiency syndrome
ALT	Alanine aminotransferase (SGPT)
ANOVA	Analysis of variance
AP	Alkaline phosphatase
ARDS	Acute respiratory distress syndrome
AST	Aspartate aminotransferase (SGOT)
AUC	Area under the plasma concentration versus time curve over time periods: AUC_{0-24}, $AUC_{0-\infty}$
BAL	Bronchial alveolar lavage
bpm	Beats per minute
BMI	Body Mass Index = Body weight (kg) / Height (m^2)
BP	Blood pressure
BW	Body weight
CFU	Colony forming unit (10^x /mL)
CAP	Community acquired pneumonia
Cl	Total clearance
C_{max}	Maximal plasma concentration
CPIS	Clinical Pulmonary Infection Score (see Appendix I)
CPK	Creatine phosphokinase
CRP	C-reactive protein
CRF	Case Report Form
C_{ss}	Plasma concentration at steady state
CV	Coefficient of variation
C_{xh}	Plasma concentration at end of infusion
DBP	Diastolic Blood Pressure
DR	Dose response
ECG	Electrocardiogram
ED_{50}	Effective dose at 50% of maximum pharmacological effect
ELF	Epithelial lining fluid
ERC	Ethical Review Committee
FI_{O2}	Percentage of inspired oxygen
Gamma-GT	Gamma glutamyltransferase (γ-GT)

GCP	Good Clinical Practice
GFR	Glomerular Filtration Rate
GI	Gastrointestinal
Hct	Hematocrit
HIV	Human Immunodeficiency Virus
HPLC	High Pressure Liquid Chromatography
HR	Heart Rate
i.v.	Intravenous
IC_{50}	Inhibitory concentration eliciting 50% of maximum effect
ICH	International Committee for Harmonization
ICU	Intense Care Unit
MAC	*Mycobacterioum avium* complex
MBP	Mean blood pressure (1/3 SBP + 2/3 DBP)
MCH	Mean corpuscular hemoglobin
MCHC	Mean corpuscular hemoglobin concentration
MCV	Mean corpuscular volume
MIC	Minimum inhibitory concentration
N	Number of observations
Pa_{O2}	Partial pressure of arterial oxygen
Pa_{O2} / FI_{O2}	Oxygenation
PBS	Protected brush specimen
PC	Predefined change
PD	Pharmacodynamics
PEEP	Positive end-expiratory pressure
PK	Pharmacokinetics
PT	Prothrombin time
QT	Duration between Q wave and T wave on the ECG (expressed in ms)
QTc	Corrected QT-interval (Bazett's formula) in ECG
RBC	Red blood cells
SAE	Serious adverse event
SBP	Systolic Blood Pressure
SD	Standard deviation
$t_{½}$	Elimination half-life in plasma
t_{max}	Time to reach maximal drug concentration in plasma
V	Volume of distribution
VAP	Ventilator associated pneumonia
WBC	White blood cells

1. INTRODUCTION AND RATIONALE FOR STUDY DESIGN

1.1 Introduction

Patients who are referred to the intense care unit (ICU) for mechanical ventilation have serious conditions, e.g. surgical complications after visceral, cardiovascular or pulmonary interventions, transplantation, multiple or thoracic trauma, acute respiratory distress syndrome (ARDS) elicited by aspiration, inhalation of chemicals, overdose of narcotics or sepsis, or deterioration of underlying disease such as congestive heart failure (CHF) or pulmonary insufficiency. These patients are very fragile and frequently develop ventilator associated pneumonia (VAP), a complication responsible for a high mortality (~40%) and for high costs due to further prolongation of their intubation period.

Pseudomonas aeruginosa is one of the three major Gram-negative pathogens associated with VAP and is especially feared. The virulence of this pathogen has been suggested to be, at least partially, due to quorum sensing, a system based on the production of diffusible autoinducer molecules which synchronize the production of virulence factors once a high cell density is achieved. This complex mechanism of virulence explained in detail below in Section 1.2 leaves very limited time to the host for mobilizing his immune system against this orchestrated attack. Quorum sensing is also responsible for the development of bacterial biofilms and for colonization of inert surfaces, a prerequisite to pneumonia. The colonization of the intubation device is believed to precede colonization of the respiratory tract, and to lead in a subset of patients to a *P. aeruginosa* VAP. Since colonization almost always precedes pneumonia, the presence of *P. aeruginosa* in lung secretions is not considered as diagnostic for VAP. In the case of clinical suspicion of VAP, state-of-the-art care requires to quantify *P. aeruginosa* on samples collected by bronchial alveolar lavage (BAL), by protected brush specimen (PBS) or by bronchotracheal aspirate, thus delaying the confirmation of the diagnosis.

Intrinsic resistance of *P. aeruginosa* to many antibiotics is well documented and the antibiotics with antipseudomonal activity cannot be used prophylactically due to the high risk of resistance emerging during treatment. Moreover no treatment can eradicate the pathogen from the intubation device as long as it is remaining in place.

Approximately 10-15% of the colonized patients will actually develop pneumonia, demonstrating a definite unmet medical need linked to a high risk in this ventilated population. A treatment which could control, or even inhibit, colonization of the intubation device might decrease colonization of the respiratory tract, thereby delaying the occurrence of pneumonia and allowing the patient to recover enough to be extubated prior to developing this threatening complication.

1.2 Quorum-Sensing

Pseudomonas aeruginosa regulates the production of several extracellular virulence factors (including LasA and LasB elastase, rhamnolipids and alkaline phosphatase) by a quorum-sensing circuitry composed of the *las* and *rhl* quorum-sensing systems [Van Delden and Iglewski 1998]. Using small diffusible cell-to-cell signals (homoserine lactones called autoinducers), namely 3-oxo-C_{12}-HSL and C_4-HSL, this complex regulatory circuit allows the bacterial population to synchronize the production of virulence factors and to produce them only once a high cell density (quorum) is attained. The importance of this circuit for the *in vivo* virulence of *P. aeruginosa* has been clearly demonstrated in animal models using defined mutants deficient in specific quorum-sensing genes [Rumbaugh et al 2000]. Quorum sensing has also been shown to be important for the development of a differentiated bacterial biofilm [Davies et al 1998]. The biofilm growth pattern is a prerequisite for efficient colonization of intubation devices leading to acute pneumonia in intubated patients [Van Delden unpublished results]. Because of the apparent central role of quorum sensing in the pathogenesis of *P. aeruginosa* infections, efforts have

been focused on this circuit as a target for drugs that would interfere with the production of virulence factors and reduce its capacity to colonize intubated patients.

The macrolide azithromycin is completely devoid of intrinsic bacteriostatic and bactericidal activity against *P. aeruginosa*. However azithromycin is able to increase in a clinically significant fashion the survival of animals challenged with *P. aeruginosa* when it is added to traditional anti-pseudomonas agents [Nicolau et al 1999]. Macrolides (erythromycin, azithromycin) have also been shown to be beneficial in chronic *P. aeruginosa* lung infections such as in the Asian panbronchiolitis [Fujii et al 1995], in cystic fibrosis in an open study in 7 children [Jaffe et al 1998], and in clinical cases [Reinert 1995]. Azithromycin use was also shown to decrease by nearly 70% the risk of infection to *P.aeruginosa* in a large epidemiology study in HIV patients [Sorvillo et al 2001]. Recently, a beneficial effect on preservation of lung function has been identified in adult cystic fibrosis patients who had been treated for three months with 250 mg oral azithromycin, using a randomized, placebo-controlled design [Walter et al 2002].These beneficial effects have been suggested to be due either to immunomodulation or to reduction of virulence factor production by *P. aeruginosa* [Howe and Spencer 1997, Peckham 2002] . It has been recently demonstrated that azithromycin interferes with the production of the cell-to-cell signals required for quorum-sensing and thereby reduces the production of extracellular virulence factors [Tateda et al 2001]. Since these signaling molecules are required for the colonization of inert surfaces, azithromycin might also reduce the capacity of *P. aeruginosa* to colonize intubated patients.

1.3 Background on azithromycin

Azithromycin, a macrolide with an azote in the lactone ring (azalide), has been selected to test this appealing mechanism of action for controlling *Pseudomonas aeruginosa* infections in humans, i.e. not by inhibiting its growth or causing death, but by reducing both its virulence and its capacity to colonize inert surfaces. The choice of azithromycin is based on substantial documentation in preclinical experiments and in infections with mutants having deficient genes, and on its excellent characteristics in terms of systemic tolerance, kinetic, metabolism (biliary excretion, low interaction potential, long biological half-life) and finally for its outstanding tissue distribution, both at interstitial and at intracellular level [33].

Azithromycin as an antibiotic acts by binding to the 50S ribosomal subunit of susceptible microorganisms, thereby interfering with their protein synthesis but without affecting their nucleic acid synthesis. It is active *in vitro* and in clinical infections against a wide spectrum of aerobic gram-positive agents (*Staphylococcus aureus, Streptococcus pneumoniae),* various staphylococci but not if methicillin-resistant and not against the majority of *Enterococcus faecalis* strains. Azithromycin shows cross-resistance to gram-positive strains resistant to erythromycin. It is not active against Enterobacteriaceae, Pseudomonas spp., or Salmonella. Despite its being a macrolide, azithromycin has however activity against some gram-negative agents such as *Haemophilus influenzae, Moraxella catarrhalis* and *Neisseria gonorrhoeae* and it is very active against some atypical microorganisms such as *Chlamydia pneumoniae* or *Legionella pneumophila* or against *Mycoplasma pneumoniae*. Minimum inhibitory concentrations for 90% of strains (MIC_{90}) are ≤0.5 µg/mL for streptococci and 2 µg/mL for other sensitive strains whereas for *Pseudomona aeruginosa* it reaches ≥128 µg/mL, a massive difference [33].

Compared to other agents, this antibiotic has unique pharmacokinetic properties, with low serum concentrations but high intracellular concentrations of the host cells such as polymorphonuclear leukocytes (PMNL), monocytes, alveolar macrophages and fibroblasts, rendering azithromycin ideal for treating tissue infection [Schentag and Ballow 1991, Reese and Betts 1996, Rapp 1998, Alvarez-Elcoro and Enzler 1999], and especially lung infections [Baldwin et al 1990, Olsen et al 1996]. The high concentrations measured within PMNL and monocytes without changing their intrinsic migrating and phagocytic properties can even be seen as a targeting tool for delivering high concentrations of drug *in situ.* Steady state is achieved after several days of treatment but remanence of these high tissue concentrations is maintained for days after cessation of therapy, another convenient situation to justify short treatment course while ensuring good patient compliance. Accumulation within *P.aeruginosa* however, albeit *in vitro*, has been shown to be in the µg/mL range, thus far away from MIC [Tateda et al 1996].

Tissue concentrations were measured in a study evaluating the pulmonary pharmacokinetics of azithromycin (12 to 96 hours) after a single 500 mg oral dose in volunteers, giving 48 hours after dosing 3.9 µg/mL in bronchial mucosa, 2.18 µg/mL in epithelial lining fluid (ELF), and 1.56 µg/mL for sputum, 30-fold the serum concentration at the time [Baldwin et al 1990]. This 500 mg oral dose is equivalent to 185 mg administered i.v., assuming a 37% absolute bioavailability [33].Twelve hours after dosing sputum was 2.9 µg/mL. When administering 500 mg on Day 1 and 250 mg from Day 2 to 5 orally to healthy volunteers before bronchoscopy and BAL, ELF concentrations increased from 0.45 µg/mL on Day 1 (4 hrs) to 3.12 µg/mL on Day 5 (4 hrs) to decrease to 0.61 µg/mL 52 hours after last treatment, with a 58-fold ratio to serum [Olsen et al 1996].

The pharmacokinetics profile and metabolism after i.v. administration has been well reviewed [Rapp 1998, Garey and Amsden 1999, Ref 33]. After i.v. administration of 500 mg over 1 hour in patients with CAP, a Cmax of 3.63 µg/mL and a Cmin of 0.20 µg/mL was achieved in plasma on Day 5. In volunteers treated with the same dose over 5 days, a Cmax of 1.13 and a Cmin of 0.18 was measured on Day 5, with an 8% increase in Cmax, a 3-fold increase in Cmin, and a 61% increase in AUC vs. Day 1. Terminal serum half-life is ~60 hours but biological half-life lasts for days. Metabolism is very limited with excretion occurring mainly unchanged via the feces, urinary excretion being very minor (6% after oral dose, 14% after i.v. at steady state). Thus no dose adjustment is required for patients with class A and B cirrhosis and in patients with renal insufficiency, a major advantage [Ref.33]. Protein binding in serum is dose dependent in the range seen under human exposure, decreasing from 51 % at 0.02 µg/mL to 7% at 1 mg/mL.

No major drug interactions are noted with theophylline, midazolam, cimetidine or zidovudine, a major advantage over clarithromycin and erythromycin. Care is recommended with associated anticoagulants, ergotamine derivatives, triazolam (decreased clearance). Drugs metabolized by the cytochrome P450 pathway e.g. carbamazepine, cyclosporine, hexobarbital, phenytoin, may have increased serum levels [Rapp 1998, Ref 33].

Clinically, azithromycin is given orally as single dose (500 mg or 1000 mg for GU infections), or as loading dose (500 mg) followed by 2 to 4 maintenance doses (250 mg) for upper respiratory tracts infections, usually 3 days for sinusitis and 5 days for community acquired pneumonia. The iv formulation has been used mainly to initiate therapy in seriously ill patients (250 mg/day or 500 mg/day) before switching to oral therapy, or to treat patients unable to take drug orally, usually for 2 to 5 days over 7-10 days of total treatment [Ref 33, Garey and Amsden 1999]. Higher doses and longer treatment durations have been explored however as described under Section 1.4.

Tolerance in Phase III programme combining i.v. route (500 mg) and later oral route (250 or 500 mg) is good, with GI complaints being the most frequent adverse events in CAP patients (total ~12%), especially diarrhea, nausea, abdominal pain, and vomiting but also occasional headache, dizziness, and rash (1-3%). Ototoxicity is a rare event at these doses. If 500 mg iv for a few days is continued with 500 mg orally, the overall GI symptoms in CAP patients are higher (~36%) [Azithromycin i.v., NDA 50,733, Clinical Section, January 1997]. Liver enzymes are occasionally increased during therapy. Long term treatment with azithromycin, over weeks and even months, has been has been gathered orally with a good safety profile. The systemic tolerance profile is similar for oral and iv. Locally the iv formulation is reasonably well tolerated, with 12 % complaints, pain (6.5%) and inflammation (3.1%) being the more frequent. Infusion over 1 hour (2 mg /mL) elicits more complaints than over 3 hours (1 mg/mL), an event possibly linked to concentration and probably decreased when given via a central line [Ref 33].

Twenty-four volunteers have received single i.v. infusions with higher doses (placebo, 1000, 2000 or 4000 mg) [Luke et al 1996]. Local and systemic tolerance was good, with occurrence of abdominal cramps at 2 and 4 g. Pharmacokinetic parameters across doses were dose dependent, with an elimination half-life of 69 hours and a 33.3 L/kg volume of distribution at steady state, reflecting the extensive tissue and cell distribution.

More information on preclinical profile, on clinical experience with azithromycin administered as an antibiotic, and on safety profile after i.v. route can be gathered in the Zithromax® Package Insert for i.v. formulation dated January 2001 [Ref 33] and the Investigator Brochure dated October 2002.

1.4 Rationale for Study and Dose Selection

Based on the scientific hypothesis exposed above and the preclinical evidence *in vitro* and even *in vivo* with animals infected with mutants, the scientific and medical rationale for this exploratory Proof-of-Concept Phase IIa appears warranted. The mechanism of action is thought to involve blocking of quorum sensing, and not to function over any direct bacteriostatic or bactericidal activity by inhibiting bacterial protein synthesis. The levels obtained albeit *in vitro* [Tateda et al 1996] show a dose and a time dependent intracellular accumulation within *P. aeruginosa*, raising from 0.190 µg/mL at 12 hours, to 0.445 at 24 hours, and to 1.709 at 36 hours when grown on agar with 10 µg/mL azithromycin; although it is expected to significantly accumulate within *P. aeruginosa* cells in clinical situations, the levels achieved should remain markedly lower than the identified MIC of ≥128 µg/mL over the 24 hours of treatment. The i.v. route is essential in this intubated patient population and ensures well controlled plasma levels and high tissue concentrations which lead to a sustained bronchial mucous concentration: 2 µg/mL is the target level documented *in vitro* and *in vivo* and it should be maintained for at least 12 hours. A daily dose of 300 mg has been selected for this study for iv administration to ensure achieving this goal; based on a 30-fold ratio sputum to serum [Baldwin et al 1990] ¨and assuming a serum through concentration around 0.12 µg/mL at steady state: the bronchial mucous level should be ~4 µg/mL throughout dosing intervals.

In patients daily i.v. doses of 500 mg are usually administered for 1 to 3 days before switching to oral formulation, occasionally for 5-6 days in hospitalized patients with CAP across a broad age range [Ref 33]. Single iv doses of 1 single i.v. infusions with higher doses (placebo, 1000, 2000 or 4000 mg), were well tolerated with occurrence of abdominal cramps at 2 and 4 g [Luke et al 1996]. In other studies, i.v. doses up to 1000 mg and up to 2 mg/mL for 10 days were well tolerated [Azithromycin for i.v., NDA 50,733, Section Clinical Pharmacology and Biopharmaceutics, January 1997]. Local tolerance at infusion site is not dose dependent but concentration dependent whereas systemic tolerance, mainly GI events, is clearly dose dependent. The dose selected in this study (300 mg daily, 1.2 mg/mL over 1 hour) is actually lower than the recommendations for community acquired pneumonia (CAP) in the initial phase [Ref 33] but the treatment will be maintained for a longer period of time, for a maximum of 20 days. It should be stressed that long term tolerance data with oral azithromycin has been gathered for 3 months in cystic fibrosis at 250 mg [Jaffe et al 1998; Walter et al 2002], for 1 to 3 months in post myocardial infarction patients with elevated *Chlamydia pneumonia* antibodies - a situation hypothetized to be involved in progression to atherosclerosis - in 3 studies at 250 mg or 500 mg daily then weekly [Gupta et al 1997; Jackson et al 1999; Anderson et al 1999] but also in a large scale ongoing trial involving thousands of patients (WIZARD study). Finally, high oral azithromycin doses have been given to AIDS patients for treatment of disseminated *Mycobacterium avium* complex (MAC): 600 mg for 4 months [Ward et al 1998] but also at 600 and 1200 mg daily for 6 weeks [Koletar et al 1999]. At these high daily doses, occurrence of GI symptoms (diarrhea, nausea, vomiting, abdominal cramps) was high (61%) although usually mild at 600 mg whereas it was even higher (79%) with moderate to severe intensity at 1200 mg [Koletar et al 1999]. A 1200 mg oral dose corresponds to 450 mg i.v. if assuming 37% bioavailability for systemic exposure, but it could correspond to approximately 600 mg i.v. if the lower serum protein binding is taken into consideration. For the intestinal load however, and as a consequence of high fecal elimination both for the oral (94%) and for the i.v. route (86% at steady state) [Ref 33], the situation is quite different: a 600 mg oral dose would be excreted via feces as 564 mg, and 1200 mg as 1128 mg. In contrast, the 300 mg i.v. dose proposed for this study would be recovered in the feces as 258 mg, comparing well with the 235 mg eliminated by a classical 250 mg oral dose. Thus the occurrence of GI adverse events in this study when using a 300 mg i.v. dose daily should be in an acceptable range while the systemic exposure would remain within the already explored concentrations measured in MAC patients.

Beyond classical bacteriological control in tracheal aspirate for colonization, sophisticated *in vivo* and *in vitro* methods to measure levels and production of autoinducers and virulence factors will be used among other parameters to document and demonstrate the mechanism of action, i.e. quorum-sensing blockade.

2. STUDY OBJECTIVES

Primary objective

- To assess the clinical efficacy of azithromycin 300 mg i.v., used as a quorum-sensing blocker, when compared to placebo for preventing or delaying the occurrence of pneumonia in ventilated patients colonized with *Pseudomonas aeruginosa*

Secondary objectives

- To assess other clinical efficacy variables
- To compare safety profile in target population
- To demonstrate using both *in vivo* and *in vitro* parameters whether azithromycin can be used clinically as a quorum-sensing blocker against *P. aeruginosa*
- To determine whether *P. aeruginosa* can develop resistance to the quorum-sensing blocker effect of azithromycin
- To check whether prolonged treatment with azithromycin may induce resistance to any of 11antipseudomonal antibiotics
- To verify whether emergence of resistance to other bacteriae may be elicited by assessing rate of infections in both study groups (azithromycin vs. placebo)

3. STUDY DESIGN

This multicentre study follows a 2-parallel group, double blind, placebo controlled design, with daily i.v. administration of 300 mg azithromycin or saline (vehicle) over 1 hour. The duration of treatment for each mechanically ventilated patient will be for a maximum of 20 days (or earlier if extubation, if occurrence of pneumonia, or if exitus).

4. SELECTION OF SUBJECTS

4.1 Number of Subjects

Recruitment will be stopped after 10 cases of pneumonia to *P. aeruginosa* have been documented in this multicentre study and confirmed by a panel of experts maintained blinded. Based on a 10% occurrence of pneumonia in patients colonized by *P.aeruginosa* in the placebo group, the total number will range between 100 and 200 assessable patients, or between 120 and 240 if applying a 20% dropout rate. The total number of patients recruited will actually depend on the precise % of patients developing pneumonia in the placebo group in each centre and on the degree of clinical efficacy seen in the azithromycin treated group (for more details, see Section *11.5 Justification for Study Size*).

4.2 Inclusion Criteria

Subjects meeting all of the following criteria will be considered for admission to the study:

- Male and non pregnant female aged 18 to 75 years
- Patients hospitalized in ICU, under mechanically assisted ventilation expected to be mandatory for 3 days or more
- Reasonable survival chance within next few days with an Apache score 10-25 as per Appendix H [Knaus et al 1985]
- Tracheal aspirate found positive to *P. aeruginosa*
- The subject (or a close family member in case of incompetence) understands the procedures, agrees to participate, and is willing to give his written informed consent

Informed consent must be obtained for all subjects before enrollment in the study, by patient or by close family member.

4.3 Exclusion Criteria

Subjects presenting with any of the following will not be included in the study:

- Poor prognosis as judged by Apache II score >25
- Pregnant females
- Grossly under- or overweight (BMI <18 or >29)
- Ongoing therapy with a macrolide (clarithromycin, erythromycin, azithromycin, telithromycin)
- Known allergy to any macrolides
- Proven *P. aeruginosa* pneumonia (with documented CFU>10^4 in BAL or PBS)
- Ongoing antipseudomonal therapy with a susceptible colonizing strain as proven by antibiogramme*

---

* A list of recommended antibiotics deprived of antipseudomonal activity is provided in Section 6.2 to facilitate the choice of the investigators and to homogenize antibiotic treatment according to type of infections. For patients on antibiotherapy with activity against pseudomonas prior to study entry, they will be maintained in the screening phase for 48 hrs, pending results of antibiogramme on the colonized strain. If found to be susceptible to the antibiotic used, the patient will be excluded from the study; if found to be resistant, the patient will be included in the study (also see Section 6.2)

- Anticipated short duration of mechanical ventilation (≤3 days)
- Known drug interaction which could interfere enough with absorption, metabolism, or excretion and thus could decrease efficacy or raise safety concern despite plasma drug monitoring of concomitant therapy (for examples see Section 6.2)
- Severe hepatic insufficiency (type C i.e. score >10 on Child Pugh scale, Appendix A) [Pugh et al 1973]
- Sick sinus syndrome or known long QT syndrome or QTc >500 msec
- Recent donation of blood or participation in another clinical trial within 3 months (Europe) or 1 month (USA)
- Any situation exposing the patient to higher risk or possibly confounding results of the study

Any local or country specific regulations must be respected. Any waiver of the inclusion and exclusion criteria listed above must be approved by the investigator and by the monitor on a case-by-case basis prior to enrolling the subject. This must be documented by both the monitor and the investigator.

No subject will be allowed to enroll in this study more than once if study drug was administered. However a patient may be screened on a 2nd occasion under another screening number if he did not meet all inclusion and exclusion criteria in the first instance.

5. STUDY TREATMENTS

5.1 Details of Study Treatments

Treatment

Drug code	ANB 006
INN	Azithromycin (dihydrate)<br>MW 785.0
Dosage form	Glass vials, 10 mL, sterilized<br>Lyophilized azithromycin 500 mg<br>Inactive ingredients (413.6 mg citric acid and sodium hydroxide)<br>Under vacuum
Route	i.v. solution in sterile 0.9% sodium chloride, 250 mL, in sterile pouch<br>Administered as 1 hour infusion (1.2 mg/mL) after reconstitution in 2 steps<br>For details, see Appendix C
Dose	300 mg
Duration	Multiple dose, once a day, for a maximum of 20 days
Manufacturer	Ben Venue Laboratories Inc.<br>Single commercial batch USA<br>The batch number and the expiration date of lyophilized material will be recorded in the Clinical Study Report.
Clinical supplies	No specific release required since commercial batch used without re-packing<br>Study label printed and affixed by Quintiles on each vial

Placebo

Dosage form	Not applicable
Dose	0 mg
Route	i.v. solution of sterile 0.9% sodium chloride, 250 mL, in sterile pouch<br>Administered as 1 hour infusion after reconstitution in 2 steps<br>For details, see Appendix C
Manufacturer	To be purchased locally by each local hospital pharmacy as a single batch<br>The source, batch number and the expiration date of lyophilized material will be recorded in the Clinical Study Report.

Subjects will be administered i.v. from infusion pouches as a solution of 250 mL containing 300 mg azithromycin in saline at 1.2 mg/mL or normal saline (placebo), over 1 hour.

Vials with lyophilized material can be stored at room temperature (30 °C or 86 °F and below). After reconstitution, azithromycin solutions (100 mg/mL and 2 mg/mL) can be stored for 24 hours at or below room temperature (30 °C or 86 °F) or for 7 days if stored under refrigeration (5°C or 41°F).

5.2 Dosage Schedule

At each study site, the study medication will be prepared by the hospital pharmacist, using the method described in Appendix C, the randomization numbers allocated to the site and the randomization code provided by the independent statistician. The pouches will be infused i.v. by the investigator or his delegate, carefully following the procedures set out in this study protocol. Administration of study drug will be time zero, Day 1 (i.e. t=0 start of infusion) and the i.v. infusion will last 1 hour. Exact date, start

and completion time of infusion will be recorded daily in the case report form (CRF). If a central line is available, the study drug will be preferably infused by that port, avoiding any other concomitant infusion.

Efforts will be made to comply with a 24 hour interval between daily doses for a given patient throughout the treatment period as much as technically feasible. The maximum duration of treatment will be 20 days.

5.3 Treatment Assignment

The study medication will be administered only to subjects included in this study following the procedures set out in the study protocol.

Since the screening period is expected to be short, no dedicated **subject numbers** will be allocated in advance but only a simplified code combining centre number (1, 2, 3, …), letter S (for screening), and the chronological number of entry (1,2,3,…). Thus, in Centre 5, the 11th patient screened would have the subject number **5-S11**, and, in Centre 12, the 2nd patient screened would carry the subject number **12-S2**. This subject number will be used to identify the subject between initiation and completion of screening, with verification of inclusion/exclusion criteria, and on Day -1. Thereafter, starting on Day 1, each subject will be assigned a **randomization number,** both sequentially and in chronological order, which will combine the study site number and a study medication number i.e. #05108 for centre 5 and 8th patient. Each patient will receive the corresponding study medication (placebo or azithromycin), labeled with that same code number. The investigator will record in the CRF the subject screening number and the 5-digit randomization number.

The study medication will be randomly assigned in advance to the randomization numbers taken into consideration the study site (#01101 to 01124 for centre 1) for a maximum of 25 study sites although 20 sites are targeted initially, according to the randomization plan generated by the Biometrics Department of Quintiles for each site and following a balanced basis (equal groups), by blocks of 4. Each centre will have a different allocation schedule. Each centre will initially receive the number of azithromycin vials corresponding to treatment for 25% of committed patients assuming 20 days of treatment (2 boxes of 10 vials per patient) and taking into account the anticipated patient recruitment (8, 12, 16, 20,…) Additional vials will be shipped to each centre based on recruitment and duration of treatment. Supplementary azithromycin vials will be maintained at disposal of centers recruiting beyond original plans or for new centres in case of need. The randomization plans will be stored with the Biometrics Department. Subjects withdrawn from the study will retain their subject number and their randomization number if drug has already been administered. New subjects must always be allotted a new subject number.

Study medication will be prepared by the hospital pharmacist in accordance with the randomization lists prepared by an independent statistician. All randomization schedules will be stored with the hospital pharmacist. The investigator and the ICU will receive sealed individual envelopes (see below Section 5.4).

5.4 Blinding, Packaging, and Labeling

The vials with lyophilized azithromycin will be stored at room temperature (30 °C or 86 °F) or below, under lock, pending preparation by the study site hospital pharmacist, according to study allocation schedule and detailed procedures described in Appendix C procedures for dilution in 2 steps. The investigator will provide the pharmacist in a timely fashion with a precise schedule for treatment days for a given subject (initials and code #) to be treated. Dilution of active drug will be performed with sterile saline 0.9 % purchased at study start by hospital pharmacy (same batch). Placebo will be prepared with the same sterile saline solution. The infusion pouches will be delivered to the investigator at regular intervals during the period as considered most convenient for pharmacy and clinical site (daily, 1, 2 or 3 times a week), providing storage temperature is respected (room temperature up to 24 hours, 5 °C or 41 °F up to 7 days until 1 hour prior to administration). The code will not be discussed or alluded to by the pharmacist, either when discussing with the investigator and his staff, the patient or the monitor to maintain the double blind conditions.

The twin study labels (multilingual as required) for the clinical supplies will be printed for each subject and each study day by Quintiles, the left side label being attached to the infusion pouch by the pharmacist, and the right side label being later detached and affixed to the CRF. The expiry date provided on the labels of both study drugs (azithromycin, saline) will correspond to the date of administration (7 days if pouch stored at 5°C, 1 day if stored at room temperature) and not to the azithromycin batch expiry date. These labels will contain the following information:

Study labels for distribution to investigator

To be attached on pouch	To be attached on CRF
**ANB 006 #2001**<br>**Azithromycin or Placebo for i.v. infusion**<br>*Store 5 °C or 41 °F, do not freeze, 250 mL*<br>Drug exclusively for clinical trial<br>Regulatory text (country specific)<br><br>Allocation # ……. Anbics<br>Day 1, 2, 3,……… Zurich<br>Expiry date……… Batch……..	**ANB 006 #2001**<br><br>Allocation #<br>Day 1, 2, 3,…<br>Date dispensed……..<br><br>Anbics<br>Zurich<br>Batch................

5.5 Supplies and Accountability

The site pharmacist will inventory and acknowledge receipt of all shipments of study medication which must be kept. at room temperature or below, pending preparation and dilution as stated above under Section 5.4. and in Appendix C.

All study medication must be kept in a locked area with access restricted to designated study personnel. The investigator and pharmacist will also keep accurate records of the quantities of study medication prepared and dispensed. During the study the monitor will inquire at regular intervals (such as every 4 patient block) on number of available azithomycin vials. The site monitor will check the supplies of study medication held and dispensed by the pharmacist and administered by the investigator to ensure accountability of all study medication used, but only after study completion, after the database has been frozen, and the code has been unblinded. At the conclusion of the study, all unused study medication and all medication containers will be destroyed locally under appropriate control and documentation unless other arrangements have been approved with the sponsor's representatives. A final report of drug accountability as agreed with the site monitor will be prepared and maintained by the pharmacist.

5.6 Compliance

The study medication will be administered by the investigator or the subinvestigators.

6. PRIOR AND CONCOMITANT ILLNESSES AND TREATMENTS

6.1 Prior and Concomitant Illnesses

Since this study will be performed in severely ill patients requiring mechanical ventilation, concomitant illnesses, postsurgical complications, or multiple trauma are expected in this population and can be among others:

- Deterioration of underlying diseases such as congestive heart failure or pulmonary insufficiency
- Development of acute respiratory distress syndrome (ARDS) due to aspiration, inhalation of chemicals, overdose of narcotics, or sepsis
- Thoracic or multiple trauma
- Complications after visceral, cardiovascular, pulmonary surgery or post transplantation

6.2 Prior and Concomitant Treatments - Antibiotherapy

Any treatment that are given in addition to the study treatment during the study is regarded as a concomitant treatment and must be documented on the appropriate pages of the CRF and in the subject's medical records, with dose adjustments as and if required. It is expected that a broad spectrum of concomitant treatments will need to be administered to treat the basic underlying disease or condition of these patients, to alleviate their symptoms, or to anticipate possible complications.

This may include antibiotics to treat infections, whenever possible selecting however an agent deprived of activity against *P. aeruginosa* or having demonstrated lack of activity against the specific strain isolated in that given patient. It is recommended to select an antibiotic to be used prior to or during the study for intercurrent infections from the table provided below to facilitate the choice of the investigators, to homogenize antibiotic treatment according to type of infections, and to limit interference with *P. aeruginosa* activity; one of these antibiotics should be given preferentially for empiric as well as for definitive therapy unless the clinical indication and/or resistance profile would justify another antibiotherapy.

Type of Infection	Recommended Antibiotics Deprived of Antipseumonal Activity
**Vascular line infections**	Methicillin, flucloxacillin, vancomycin, teicoplanin, synercid
**Abdominal infections**	Ceftriaxone +/- metronidazol
**Urogenital infections**	Ceftriaxone +/- metronidazol<br>Amoxicillin + clavulanic acid +/- metronidazol
**Pulmonary infections**	Cetriaxone<br>Amoxicillin + clavulanic acid
**Central nervous system infections**	Ceftriaxone, vancomycin
**Skin infections**	Penicillin, vancomycin, teicoplanin<br>Amoxicillin + clavulanic acid

The following antibiotics listed in the table below have antipseudomonal activity and should be avoided for therapy unless the specific colonizing strain happens to be resistant or if mandatory during the study for clinical reasons. These same 11 antiobiotics will be screened for MIC on bronchotracheal aspirate at study completion for assessing emergence of resistance.

**11 Standard Antipseumonal Antibiotics Not Recommended for Clinical Use* To Be Tested for MIC Pattern**	
Carbenicillin	Ceftazidime
Piperacillin	Gentamycin
Piperacillin + tazobactam	Tobramycin
Imipenem	Amikacin
Ciprofloxacine	Meropenem
Cefepime	

* Except if isolated *P. aeruginosa* strain resistant or if medically indicated

Patients on antibiotherapy with activity against pseudomonas prior to study entry will be maintained in the screening phase for 48 hrs, pending results of the antibiogram on the colonized strain. If found to be susceptible to the antibiotic used, the patient will be excluded from the study; if resistant, the patient will be included in the study. Patients already included in the study but requiring an antibiotic with activity against the colonizing *P.aeruginosa* strain will be maintained in the study. Statistical analysis will be performed in these patients as a subgroup and combined with remaining patients only if the pattern of response between treatments is found to be similar to patients treated with no antibiotics or with antibiotics deprived of antipseudomonal activity.

When suspicion of *P.aeruginosa* pneumonia is raised by the clinical situation, a diagnostic procedure must be performed (BAL, PBS) before an antibiotic with antipseudomonal activity can be given empirically. In the case *P.aeruginosa* pneumonia has been diagnosed (Day x) by quantitative culture, dedicated antibiotherapy to treat *P.aeruginosa* infection is recommended and the patient should be taken out of the study at that stage.

Except for other macrolides no treatment is forbidden during the study as reflected in the inclusion/exclusion criteria. Few clinically significant drug interactions are expected to impact on azithromycin clearance, metabolism or distribution and thus concomitant therapy should not influence the clinical outcome of this study. However it is prudent to monitor plasma concentration of concomitant drug which could be increase, thus leading to toxicity, when administering:

- Drugs such as theophyllin, digoxin, ergotamine, dihydroergotamine, or triazolam
- Compounds metabolized by cytochrome P450 system such as carbamazepine, cyclosporine, hexobarbital or phenytoine
- Drugs known to increase QTc wave such as selected H1-antihistaminics
- During therapy with anticoagulants such as warfarin should be monitored regularly by prothrombin time as performed in this study

Dosages of concomitant drugs should be adapted for the best interest of the patient if required for efficacy and/or safety reasons.

7. STUDY PROCEDURES AND SCHEDULE

7.1 Overview of Data Collection

The schedule of data collection is described in the Study Schedule and in Study *Section 7.2. Description of study days*; the methods of data collection and the analysis variables are described in *Section 7.3. methods of data collection* and in the appropriate Appendices. Preparation of clinical supplies is described in *Section 5.4* and in *Appendix C: Preparation of i.v. supplies*. Prior and concomitant therapy as well as antibiotherapy recommended are described in *Section 6.2*

7.1.1. Clinical efficacy data (collected at each clinical site)

Clinical efficacy (clinical site)

- Occurence of *P. aeruginosa* pneumonia (primary variable)

  The diagnosis of pneumonia will be assessed under blinded conditions by an independent panel of experts prior to stopping the study
- Occurrence of death (excluded if death within 3 days and not elicited by *P. aeruginosa*)
- Time to pneumonia, to exitus (Day –1), or to bacteremia
- Time to extubation (Day n-1)
- Overall outcome (until Day n+7)
- Duration of hospital and ICU stay
- Cost assessment
- Pa_{O2} / FI_{O2}
- Occurrence of infections to other bacterial strains

7.1.2. Pharmacodynamic data performed in local laboratory

A. Tracheal aspirates (on Aspirate 1)

- Collection daily
- Quantitative culture for *P. aeruginosa* **(**CFU/mL) on Day –1, 1, 3, 6, 9, 12, 15, 18 and last day (Day x, or Day n-1, or Day 20)
- Isolate *P. aeruginosa* strain daily and store at –70°C on skim milk and glycerol
- Emergence of resistance (MIC to *P. aeruginosa* in tracheal aspirate for 11 standard antipseudomonal antibiotics on Day –1 and on last day (Day x, or Day n-1, or Day 20) see list under Section 6.2)
- CFU for other strains from tracheal aspirates if indicated

B. BAL on Day x (or protected brush specimen (PBS) or bronchotracheal aspirate)

- Only for patients with suspected pneumonia for diagnosis
- CFU/mL
- Cellularity

7.1.3 Pharmacodynamic data performed in Geneva laboratory

A. Tracheal aspirates (*ex vivo,* on Aspirate 2)

- Collection daily at study site, storage at –70°C prior to transfer to Geneva
  - Day -1, 1, 3, 6, 9,…, last day (Day x, or Day n-1, or Day 20) for all patients
  - Daily for pneumonia (n=10) and control patients (n=20)
  - Daily for patients on antibiotherapy
- Parameters
  - Content for autoinducer homoserine lactones: 3-oxo-C_{12}-HSL and C_4-HSL **and/or**
  - mRNA for LasB elastase (lasI, rhlI)

B. Respiration device on Day n

- CFU/cm^2
- Autoinducer content

C. Isolated *P.aeruginosa* strains from tracheal aspirate 1 and respiration device (*in vitro*)

- Day -1, 1, 3, 6, 9,…, last day (Day x, or Day n –1, or Day 20) for all patients Daily for pneumonia (n=10) and control patients (n=20) selected on a site basis
- Daily for patients on antibiotherapy
- *In vitro* virulence factor production (elastase, protease, rhamnolipids, cytotoxicity)
- *In vitro* autoinducer production (3-oxo-C_{12}-HSL and C_4-HSL)
- Azithromycin gradient plates for detection of rhamnolipid production (Day -1, or Day x, or Day n-1) to detect possible resistance mechanisms

7.1.4. Safety data

- Adverse event profile (reported by subject, observed by investigator)
- Local tolerance (infusion site if peripheral site)
- Standard laboratory tests (see Appendix B)
- Blood gases
- Vital signs (blood pressure, heart rate, body temperature)
- 12-lead electrocardiogramme (ECG)
- Emergence of non Pseudomonas infections

7.2 Description of Study Days

7.2.1 General Recommendations and Procedures for Study

Test drug solutions (Azithromycin or placebo saline as required) will be prepared by the site pharmacist (see Section 5.3, 5.4 and Appendix C) in adequately labeled pouches transmitted to the investigator for storage at 5 °C or 41 °F until 1 hour prior to use. The temperature of the solution must be close to room temperature when infused. The same peristaltic pump should be used throughout the study. The exact

time of start and completion of the infusion will be recorded in the CRF, along with the volume infused (250 mL) and the label. It is not planned to collect retain samples pre or post infusion in this study. Efforts will be made to rotate infusion sites if a peripheral vein is used every 4th or 5th day, or more often in case of local signs. Preference will be given to a central line if available to ensure high blood flow.

Time (T=0) is always calculated from start of test drug infusion. Cannulae will be maintained patent with a slow saline infusion. No other drug may be infused concomitantly using the same i.v. line. A 24 hour time frame between daily infusions should be attempted.

Clearly a **hierarchy** for activities must be followed, collecting predrug (0 hr) samples (tracheal aspirate 1, blood safety, blood for WBC, blood gases, and blood culture if required etc.) or predrug information (vital signs i.e. blood pressure and heart rate, oxygenation, systemic and local tolerance, fever), all before initiating the test drug infusion. The precise time for starting and stopping the test drug infusion, for collecting tracheal aspirate 1 (predrug), aspirate 2 (~6 hours post infusion start), should be recorded in the CRF. ECG will be recorded 1 to 4 hours post infusion start as indicated in the appropriate study schedule and following recommendations of Appendix J. Fever will be measured in late afternoon (around 17:00 o'clock) and vital signs 6 hour post test drug infusion start (around 15:00 o'clock). Systemic and local tolerance will be observed throughout the study, until a week after cessation of therapy for adverse events for the former (i.e. Day n+7).

7.2.2 Prestudy Screening (Day -1)

The following examinations and tests may be carried out over 2 or occasionally 3 days as required for obtaining results or confirmation for each subject before entering the study:

- Check likely adequacy of inclusion/exclusion criteria
- Explain study to the patient (or to a close family member if not conscious or not in a position to make a decision) and obtain written approved consent
- Confirm inclusion/exclusion criteria with strong emphasis on inclusion criteria (assisted ventilation anticipated to last for at least 3 more days, colonization of tracheal aspirate by *P. aeruginosa*, APACHE II score between 15 and 25, etc.)
- Assign a screening subject number
- **Record:**
  - Complete physical examination and medical history, including age, sex, and race
  - Height and weight (from patient dossier)
  - Blood pressure, heart rate, respiration rate (8:00, 14:00)
  - Body temperature (tympanic or oral) 7:00 (and 17:00 if selected)
  - Blood (see Appendix B)
    - Hematological profile
    - Blood chemistry
    - WBC & CRP
  - Urinalysis screening (see Appendix B)
  - 12-lead electrocardiogramme
  - Blood gases
  - Oxygenation: Pa_{O2} / FI_{O2}
  - Lung X-Ray

- Apache II score
- CPIS
- Tracheal aspirate 1 (~8:00) for transfer to local laboratory (for parameters see Section 7.1.2)
  - Quantification of *P. aeruginosa* (CFU/mL)
  - Isolation of *P. aeruginosa* strain (storage –70°C)
  - Special Day n-1 assessment for MIC on *P. aeruginosa* strain with 11 antipseudomonal antibiotics (listed under Section 6.2)
- Aspirate 2 (~14:00) for storage at –70°C
- Clinical endpoints

7.2.3 Study days

Day 1

On the morning of Day 1, prior to infusion of test drug, the following procedures will be performed:

- Adverse events
- Body temperature (tympanic or rectal) ~7:00
- Blood pressure, heart rate, respiration rate (~8:00)
- Blood (see Appendix B)
  - Hematological profile
  - Blood chemistry
  - WBC & CRP
- Specimen for blood culture (only if clinically indicated)
- Blood gases
- Oxygenation: Pa_{O2} / FI_{O2}
- CPIS (use X-ray of same day whenever possible, else of previous day)
- Clinical endpoints
- Tracheal aspirate 1 (transfer to local laboratory, for parameters see Section 7.1.2 above)
  - Quantification of *P. aeruginosa* (CFU/mL)
  - Isolation of *P. aeruginosa* strain (storage –70°C)

After these activities the test drug will be infused over 1 hour, usually from 8 to 9 o'clock.

Post treatment activities will include:

- ECG, 1 to 4 hours post infusion start
- Tracheal aspirate 2 (for freezing and transfer to Geneva laboratory for investigation, for parameters see Section 7.1.2 above), collected ~6 hours post infusion start
- Blood pressure, heart rate, respiration rate (~6 hours post infusion start)
- BAL if required medically (suspicion of pneumonia)

- Lung X-ray if required medically or if pneumonia (after Day x)
- Body temperature (tympanic or rectal) ~17:00
- Local tolerance (24 hours)
- Systemic tolerance (24 hours)

Day 2

On the morning of Day 2, prior to infusion of test drug, the following procedures will be performed:

- Systemic adverse events from previous night
- Local tolerance
- Body temperature (tympanic or rectal) ~7:00
- Blood pressure, heart rate, respiration rate (~8:00)
- Blood for WBC & CRP
- Specimen for blood culture (only if clinically indicated)
- Blood gases
- Oxygenation: Pa_{O2} / FI_{O2}
- Clinical endpoints
- Tracheal aspirate 1 (transfer to local laboratory, for parameters see Section 7.1.2 above)
    - Isolation of *P. aeruginosa* strain (storage –70°C)

After these activities the test drug will be infused over 1 hour, usually from 8 to 9 o'clock.

Post treatment activities will include:

- Tracheal aspirate 2 (for freezing and transfer to Geneva laboratory for investigation, for parameters see Section 7.1.2 above), collected ~6 hours post infusion start
- Blood pressure, heart rate, respiration rate (~6 hours post infusion start)
- BAL if required medically (suspicion of pneumonia)
- Lung X-ray if required medically or if pneumonia (Day x)
- Body temperature (tympanic or rectal) ~17:00
- Local tolerance (24 hours)
- Systemic tolerance (24 hours)

Day 3

On the morning of Day 3, prior to infusion of test drug, the following procedures will be performed:

- Adverse events from previous night
- Local tolerance
- Body temperature (tympanic or rectal) ~7:00
- Blood pressure, heart rate, respiration rate (~8:00)

- Blood (see Appendix B)
  - Hematological profile
  - Blood chemistry
  - WBC, CRP
- Specimen for blood culture (only if clinically indicated)
- Blood gases
- Oxygenation: Pa_{O2} / FI_{O2}
- CPIS (use results of lung X-ray of same day whenever possible)
- Clinical endpoints
- Tracheal aspirate 1 (transfer to local laboratory, for parameters see Section 7.1.2 above)
  - Quantification of *P. aeruginosa* (CFU/mL)
  - Isolation of *P. aeruginosa* strain (storage –70°C)

After these activities, the test drug will be infused over 1 hour, usually from 8 to 9 o'clock.

Post treatment activities will include:

- ECG, 1 to 4 hours post infusion start
- Tracheal aspirate 2 (for freezing and transfer to Geneva laboratory for investigation), collected ~6 hours post infusion start
- Blood pressure, heart rate, respiration rate (~6 hours post infusion start)
- BAL if required medically
- Lung X-ray whenever possible during the day
- Body temperature (tympanic or rectal) ~17:00
- Local tolerance (24 hours)
- Systemic tolerance (24 hours)

Subsequent study days in 3 day cycles (Day 4, 5 & 6; Day 7, 8 & 9; Day 10, 11 & 12; etc.)

This same treatment and sampling policy, using a 3-day cycle will be used until Day n which may correspond to Day 21 (24 hours after last treatment day i.e. after 20 days of treatment), or to extubation day because mechanical ventilation is medically not required any longer, or to exitus day (due to underlying disease complication, infection due to any strain type and site with or without sepsis, *P. aeruginosa* infections of non pulmonary location) and finally to Day x when *P. aeruginosa* pneumonia diagnosed.

The procedures to be performed daily or every third day are summarized in the Study Schedule (page 10) and as briefly reviewed below:

**Day 4, 5, 7, 8, 10, 11, 13, 14, 16, 17, 19**	**Correspond to Day 2**
**Day 6, 9, 12, 15, 18**	**Correspond to Day 3**
**Day x (pneumonia, see below)**	**Corresponds to Day 3 with some additional parameters and study drug is terminated once diagnosis confirmed**
**Day n-1 or Day 20**	**Corresponds to Day 3, and is last day of test drug (with some additional parameters)**

**Day n or Day 21**	**Discharge from study after laboratory safety and physical examination (follows Day n-1, or Day 20, or Day x whenever possible)**

If a patient has to be re-intubated within 24 hours he will remain in the study and treatment with study drug continued with 2nd extubation or until Day 20 of treatment whatever happens first. If a patient has to be tracheotomized during the study, he will remain in the study until Day 20 of treatment.

7.2.4. Day x (Day of pneumonia)

The day when a diagnosis of established pneumonia or possible pneumonia is made, will be called Day x. Treatment against *P. aeruginosa* pneumonia will be initiated as soon as the diagnosis is confirmed or if this pathogen is considered as a highly likely cause of pneumonia. The Day x procedures will be implemented as completely as possible on that day (similar to Day n-1) and the remaining items will be performed the next day (Day n). The patient will not receive the test drug on the following morning and the patient will be discharged from the study on Day n with safety and physical examination.

In case of suspicion of pneumonia the necessary explorations will be performed in order to lead to a confirmed diagnosis based on the criteria for *P.aeruginosa* summarized in the table presented below:

**Investigation**	**Documented Pneumonia Day x**
BAL<br>Or tracheobronchial aspirate<br>Or protected brush specimen (PBS)	$\geq 10^4$ CFU/mL<br>$\geq 10^5$ CFU/mL<br>$\geq 10^3$ CFU/mL
CPIS*<br>(see detailed score in App. I)	≥6

The diagnosis of pneumonia will be confirmed at later stage under double blind conditions by a panel of independent clinical experts prior to stopping the study to ensure that 10 documented cases of P. *aeruginosa* pneumonia have been included in the study for assessment. The data provided will be patient chatacteristics, symptoms, all CPIS parameters generated, CPIS overall scores, all X-rays collected and results of BAL (or tracheobronchial aspirate or protected brush). Efforts will be made to achieve a total of 10 pneumonia cases in patients who do not receive concomitant therapy with an antibiotic with activity against the isolated *P. aeruginosa* strain, providing however the overall constraint for a maximum of 240 patients entered will not be trespassed.

Post treatment activities will include:

- Tracheal aspirate 2 (for freezing and transfer to Geneva laboratory for investigation), collected early afternoon (usually 6 hours [if Day x] or 30 hours [if Day n and if applicable] after last test drug administration) if patient still intubated and even if antipseudomonal antibiotherapy has been meanwhile initiated
- ECG, 1 to 4 hours late morning Day x
- Blood pressure, heart rate, respiration rate (early afternoon Day x or Day n)
- Lung X-ray whenever possible that day

- Body temperature (tympanic or rectal) ~17:00
- Local tolerance (up to 24 hours)
- Systemic tolerance (up to 24 hours)

Physical examination and laboratory safety parameters (blood and urine) will be collected on Day n.

7.2.5 Day n-1 (last day of treatment)

Day n-1 is the last day of treatment and corresponds to:

- Day of extubation due to improvement of patient condition. If a patient has to be re-intubated within 24 hours he will remain in the study and treatment with study drug continued daily until 2nd extubation successful or until Day 20 of treatment whatever happens first
- Exitus day
- Day 20: Treatment with study drug will be stopped even if ventilation will be continued

The required tests will correspond to Day 3 requirements with additional items for discharge unless not feasible:

- Adverse events from previous night
- Local tolerance
- Body temperature (tympanic orrectal) ~7:00
- Blood pressure, heart rate, respiration rate (~8:00)
- Blood gases
- Oxygenation: Pa_{O2} / FI_{O2}
- Tracheal aspirate #1, collected pre last dose early morning if patient still ventilated for transfer to local laboratory for:
  - Quantification of *P. aeruginosa*
  - Isolation of P. *aeruginosa* strain and storage at –70°C
  - Special Day n-1 assessment for MIC on *P. aeruginosa* strain with 11 antipseudomonal antibiotics (listed under Section 6.2)
- ECG 1-4 hours post drug
- Lung X-ray whenever possible that day
- CPIS (use lung X-ray of same day)
- Clinical endpoints
- APACHE II score
- Tracheal aspirate #2 (for freezing at –70°C and transfer to Geneva laboratory for investigation), collected 6 hours post drug or earlier if extubation planned
- Extubate if applicable, if possible in the afternoon and after collection of tracheal aspirate sample 2, as medically indicated by weaning procedures
- Store ventilation device in sterile pouch at –70 °C, pending shipment to Geneva laboratory

In the case of exitus, the examinations performed on the previous day and on last third day (Day 9, 12, 15 etc.) will be used as Day n-1 values and the respiration device will be recovered and stored as described above.

On **Day n,** no study drug will be administered and the patient will be discharged from the study after physical examination and after collecting blood and urine safety samples. Ventilation will be continued if required.

7.2.6 Poststudy (Day n + 7)

Information on the patient outcome (pneumonia, infection, strain, etc.) and intercurrent adverse events over the past week will be collected one week after cessation of therapy, i.e. on Day n+7, even for patients treated with antipseudomonal antibiotherapy, whether ventilated or not. This information will be best achieved by the treating physician visiting the patient at his present location or by obtaining a report.

The Quintiles study monitor will control the labeling, storage, and coordinate the shipment of all biological samples collected during the study by the ICU nurse to the University Hospital of Geneva, Switzerland. Shipment will be performed in batches of patients when convenient for site, monitor, and Geneva laboratory (see Appendix G).

7.3. Methods of evaluation

Clinical efficacy (clinical site)

All clinical variables will be collected at the clinicial site, within the ICU, by a designated subinvestigator and ICU nurse, whenever possible by the same individuals for a given patient, barring emergencies.

Pharmacodynamic data (local laboratory)

Selected pharmacodynamic data (tracheal aspirate 1: quantitative count for *Pseudomonas aeruginosa* on Day –1, 1, 3, 6, 9, 12, 15, 18 and last day (Day x, or Day n-1, or Day 20), MIC to *P.aeruginosa* for 11 standard antipseudomonal antibiotics on Day -1 and on last day (Day x, or Day n-1, or Day 20) (see list in Section 6.2), CFU for other strains if indicated; BAL: CFU/mL, cellularity) will be performed in local bacteriology laboratory on a daily basis. MIC will be performed by one standard assay (disk diffusion assays as defined by the NCLS (National Committee for Laboratory Standards, or E-test, or dilution). All parameters will be assessed under double blind conditions for treatment.

Pharmacodynamic data (Geneva laboratory)

Extensive pharmacodynamic data both *in vivo* (tracheal aspirates 2: content for autoinducers,; mRNA for lasB elastase (lasI, rhlI); BAL: CFU/mL, autoinducer content; Respiration device: CFU/cm^2, autoinducer content) and *in vitro* (isolated strains from tracheal aspirates prepared by local laboratory, BAL and respiration device: virulence factor production, autoinducer production, azithromycin gradient plates for detection of rhamnolipid production) will be performed in the laboratory of Dr. Christian Van Delden, Department of Genetics and Microbiology, Medical School, University of Geneva, Switzerland).

This list is provided as an example only and final parameters finally agreed upon may differ and will be described in a separate document which will be produced before unblinding the study and will be attached to the final Clinical Study Report. Details on samples to be analyzed are provided in Section 7.2.3. The control patients will be selected by an independent physician who will be given access to the allocation code, pairing 2 control patients by site (1 treated with azithromycin, 1 treated with placebo) with a pneumonia patient, but not by sex or age if not achievable. All parameters will be assessed under double blind conditions for treatment allocation, but not for study day.

Laboratory safety

Hematology and clinical chemistry will be carried out according to standard operating procedures of each study site central laboratory. Predefined percentage values will be used for parameters outside normal range.

Abnormal results which do not appear to be linked to underlying pathology will be verified to rule out laboratory error. Persistent relevant abnormal values must be followed up until the cause is determined or until they return to the premedication value. Any abnormality detected at 24 hour post drug intake should be followed up until resolution or stabilization.

Clinical safety

Blood pressure and heart rate will be recorded in supine position, with sphygmomanometry or automated measurements (Dynamap or alike, central line), always using same arm, same technique, and same equipment.

Adverse events spontaneously reported or observed by the investigator will be recorded. All adverse events, including intercurrent illnesses, must be reported and documented on the CRF.

Local tolerance at test drug site will be assessed at regular intervals during each infusion (0, 1, 2, 4, 8, 24 hour or longer in case of local reaction) but only if a peripheral site is used. Any symptoms rated as moderate or severe will be reported as an adverse event. An overall grading will be used with the following scale:

0	No irritation
1	Painful site, no erythema, no swelling, no induration, no palpable venous cord
2	Painful site with erythema and some degree of swelling, or both, but no induration and no palpable cord
3	Painful site with erythema and swelling, and with induration or a palpable cord ≤7 cm above site
4	Painful site with erythema, swelling, induration, and a palpable cord >7 cm above site
5	Frank vein thrombosis in addition to grade 4 signs and symptoms

Rotating infusion sites every 4 or 5 days is encouraged to avoid problems with local tolerance. Site changes with vein involved and type of catheter (peripheral, central) will be recorded in the CRF. No local tolerance will be recorded if a central line is used. A central line will be used if available.

Twelve-lead ECG will be recorded as described in Appendix I, at a speed of 25 mm/sec and calibrated at 10 mm/mV. If a QTc is prolonged (>450 ms or >60 ms vs. baseline), manual checking of QT and calculation of QTc with Bazett's formula will be performed by the investigator for safety purposes. Manual reading will be performed at study completion by experienced cardiologist on study site. To perform centralized reading is not planned unless required at later stage.

8. ADVERSE EVENTS

8.1 Definitions

8.1.1 Adverse Event

The term **adverse event** covers any sign, symptom, syndrome, or illness that appears or worsens in a subject during the period of observation in the clinical study and that may impair the well being of the subject. The term also covers laboratory findings or results of other diagnostic procedures that are considered to be clinically relevant (e.g., that require unscheduled diagnostic procedures or treatment measures, or result in withdrawal from the study).

The adverse event may be:

- A new illness
- Worsening of a sign or symptom of the condition under treatment, or of a concomitant illness
- An effect of study medication, including test drug, placebo, and substances administered for clinical support with appropriate comment
- A combination of two or more of these factors
- No causal relationship with the study medication or with the clinical study itself is implied by the use of the term “adverse event”. Adverse events will be rated as definitely not drug related, probably not drug related, possibly drug related, probably drug related and definitely drug related, with information if test drug, concomitant therapy, underlying disease, study procedure may be involved.
- Surgical procedures themselves are not adverse events; they are therapeutic measures for conditions that require surgery. The condition for which the surgery is required may be an adverse event. Planned surgical measures permitted by the clinical study protocol and the condition(s) leading to these measures are not adverse events.

Illnesses first occurring or detected during the study such as recurrent infection or pneumonia are to be regarded as adverse events and must be documented as such in the CRF. Increase of mechanical ventilation support or changes in oxygenation will not be considered an adverse event, but the occurrence of pneumonia will be an adverse event.

Adverse events fall into the categories “non serious” and “serious” (see *Section 8.1.2 Serious Adverse Event*).

8.1.2 Serious Adverse Event

A serious adverse event is one that at any dose:

- Results in death
- Is life-threatening
- Requires prolongation of hospitalization beyond anticipation due to underlying pathology and its expected complications
- Results in persistent or significant disability/incapacity
- Is a congenital anomaly/birth defect
- Represents overdose or false route of administration (even if no adverse event has occurred)

- Is medically important

A few terms are clarified below:

- A "life-threatening" condition implies that the subject was at immediate risk of death at the time of the serious adverse event but it does not refer to a serious adverse event that could have hypothetically caused death if it had been more severe
- A "persistent or significant disability or incapacity" implies a substantial disruption of a person's ability to carry out normal life functions
- Medical and scientific judgment should be exercised in deciding whether other adverse events may be considered serious because they jeopardize the subject or may require intervention to prevent one of the other outcomes listed in the definition above. Examples of such events are intensive treatment for allergic bronchospasm, blood dyscrasias or convulsions. The List of Critical Terms (1998 adaptation of WHO Adverse Reaction Terminology Critical Terms List) should be used as guidance for adverse events that may be considered serious because they are medically important

All patients are expected to be in the ICU during the study and to remain in hospital for 2-4 weeks after study completion.

Cases involving cancer as an adverse event should be reported as "serious" using the criterion "medically important".

Due to the patient population investigated, several fatal outcomes and serious adverse event are expected in this study.

Clarification of the difference in meaning between "severe" and "serious":

The term "severe" is often used to describe the intensity (severity) of a specific event (as in mild, moderate, or severe myocardial infarction); the event itself, however, may be of relatively minor medical significance (such as severe headache). This is not the same as "serious", which is based on the outcome or action criteria usually associated with events that pose a threat to life or functioning. Seriousness (but not severity) serves as a guide for defining regulatory reporting obligations.

8.2 Period of Observation

For the purpose of this study, the period of observation extends from the time the subject gives informed consent until 7 days (Day n+7) after the last administration for tolerance and for clinical outcome.

If the investigator detects or learns about a serious adverse event in a study subject after the end of the period of observation, and considers the event possibly related to prior study treatment, he or she should contact the sponsor to determine how the adverse event should be documented and reported.

8.3 Documentation and Reporting of Adverse Events by Investigator

All adverse events that occur after the subject has signed the informed consent document must be documented on the pages provided in the CRF.

The following approach will be taken for documentation:

- **All non serious adverse events** must be documented on the "Adverse event" page of the CRF (systemic, local if considered clinically significant, biological if considered clinically significant)
- **All serious adverse events** must be documented on the "Serious adverse event form" which will be part of the CRF (basic report and updates). The information will be forwarded to the monitor and to the identified sponsor's representatives **within 24 hours, or at the latest on the following working**

day, by telephone and by Fax.. The sponsor’s representatives will ensure that all legal reporting requirements are met.

Every attempt should be made to describe the adverse event in terms of a diagnosis, and not as a list of symptoms. If appropriate, component symptoms should also be listed below the diagnosis, as comments. Non-specific signs or symptoms may be recorded as a diagnosis. Assessing the relationship to study drug or to study conditions must be performed and rating of intensity provided (definitely not, possibly, probably, not assessable).

The initial serious adverse event report must be as complete as possible, including details of the current illness and (serious) adverse event, and an assessment of the causal relationship between the event and the study medication. Information not available at the time of the initial report (e.g., an end date for the adverse event, laboratory values or investigation results received after the report) must be documented on a follow-up “Serious adverse event” form.

All subjects who have adverse events, whether considered associated with the use of the study medication or to study conditions or not, whether non serious or serious, must be monitored to determine the outcome. The clinical course of the adverse event will be followed up according to accepted standards of medical practice, even after the end of the period of observation, until a satisfactory explanation is found or the investigator considers it medically justifiable to terminate follow-up. Should the adverse event result in death, a full pathologist’s report should be supplied, whenever possible.

All questions on the completion and supply of adverse event report forms and any further forms issued to the investigator at a later date to clarify unresolved issues should be addressed to the sponsor.

Overdose is unlikely to occur in this study since the study drug (300 mg or 0 mg in 250 mL saline) is administered by the ICU staff under the investigator’s responsibility, as 1 pouch per study day prepared by the hospital pharmacist and infused i.v. at 250 mL/hr i.e. at a concentration of 1.2 mg/mL which is well tolerated. In case of overdose and from a systemic point of view, GI symptoms may occur which are related in terms of incidence and intensity to total dose given, and from a local point of view, pain and irritation at study site may occur if delivery speed would be at 500 mL/hr or above.

9. WITHDRAWALS

9.1 Withdrawal of Subjects

Patients may be withdrawn from study medication for the following reasons:

- At their own request or at the request of their legally authorized representative
- If, in the investigator's opinion, continuation in the study could represent a risk or could be detrimental to the subject's well-being
- At the specific request of the sponsor’s representatives

In all cases, the reason for withdrawal must be recorded in the CRF and in the subject's medical records. The subject must be followed up to establish whether the reason was an adverse event, and, if so, this must be reported in accordance with the procedures in Section 8.3.

As far as possible, all examinations scheduled for the final study day must be performed on all subjects who receive study medication but do not complete the study according to protocol (see Section 7.2). The investigator must make every effort to contact subjects lost to follow-up.

9.2 Replacement of Subjects

Subjects who are withdrawn for personal or family reasons, who are not assessable, who dropout for medical reasons or as a consequence of exitus within 3 days of treatment will not be replaced. Each site will try to achieve the agreed number of assessable patients.

10. EMERGENCY PROCEDURES

10.1 Emergency Sponsor Contact

In emergency situations, the investigator should contact the sponsor or sponsor's representatives by telephone using the numbers listed on the title page of the protocol.

10.2 Emergency Identification of Study Medication

If it is medically imperative to know what study medication the subject is receiving, the investigator or authorized person should open the randomization envelope. The investigator or the person who breaks the blind must record the date and the reasons for doing so in the case report form, in the subject's medical record, and on the randomization envelope. In such cases, treatment with the study medication must be stopped and the sponsor's representatives contacted immediately to determine whether the subject should be withdrawn from the study. Whenever possible, the sponsor's representatives should be contacted before the blind is broken.

10.3 Emergency Treatment

During and following a subject's participation in the trial, the investigator/institution should ensure that adequate medical care is provided to a subject for any adverse events, including clinically significant laboratory values, related to the trial. The investigator/institution should inform a subject when medical care is needed for intercurent illness(es) of which the inrvestigator becomes aware.

11. STATISTICAL PROCEDURES

11.1 Analysis Variables

All variables collected and calculated for pharmacodynamics, pharmacokinetics and safety will be analyzed (listed under Section 7.3).

11.2 Study Populations

- All subjects who are considered as available and interpretable will be included in the clinical and pharmacodynamic analysis, providing they have completed at least 3 days of treatment prior to exitus or at least 5 days prior to extubation, and providing no major protocol violations have been identified. Cases with death occurring within 3 days of entering study and apparently triggered by other causes than *P.aeruginosa* pneumonia will not be considered as assessable
- All randomized subjects who will have received at least one dose of medication and having data considered as available and interpretable will be included in the safety analysis.

11.3 Statistical Methods

The primary analysis variable will be the occurrence of pneumonia in both treatment groups (azithromycin vs. placebo), using Fischer's exact test (two-sided, p=0.10) and based on 10% in placebo group. Other clinical variables and all pharmacodynamic variables measured *in vivo* and *in vitro* will be analyzed by Fischer's test, by paired t-test, by Mann Whitney test, or by ANOVA using adequate models, as considered best indicated. The list of all clinical and pharmacodynamic variables considered is provided in Section *7.1.1*.

Clinical and Pharmacodynamic Variables

Appropriate graphs for key parameters as well as tables for all analyzed variables will be provided. A subpopulation analysis will be attempted for the primary variable and for selected secondary clinical and pharmacodynamic variables, taking into account if patients are considered as being at risk at study entry:

- Treatment with steroid or antibiotherapy
- Lung damage or transplant
- >72 hours of hospitalization prior to intubation

The variables will be analyzed for all patients and for patients without antibiotiotherapy or with antibiotics deprived of activity against the colonizing *P. aeruginosa* strain (Subgroup 1) and for patients treated with antibiotics with *P. aeruginosa* activity (Subgroup 2). If no difference between response to study drug treatment (azithromycin vs. placebo) is seen when considering Subgroup 1 and Subgroup 2, all patients will be combined. If feasible, comparisons of response taking into consideration risk factors (e.g. for subgroups with 0, 1, ≥2 risk factors) will be attempted. A detailed statistical plan will be prepared by the responsible statistician prior to decoding the study.

Clinical endpoints and outcome variables as well as pharmacodynamic variables may be used as categorical event (yes, no), as raw data (for days, concentration, score for CPIS and APACHE II score, ventilation parameters, CFU, …), as log transformed data, as differences to baseline, or as % change of baseline. All measured and calculated variables will be presented as listings by time and by treatment and, if indicated, as difference to baseline and as % change of baseline.

Graphs illustrating time effect will be provided.

Safety parameters

Adverse events

Adverse events reported during trial will be listed by treatment. Absolute and relative frequencies of subjects with adverse events (treatment emergent adverse events) will be tabulated by study part, by body system and by dose. Frequencies of subjects with severe and/or related adverse events will be summarized likewise. Local tolerance will be provided by time and by treatment and will be summarized with descriptive statistics for categorized variables by time and treatment.

Laboratory variables

Laboratory findings will be evaluated using a system of predefined changes and clinically significant abnormal values (provided by sponsor), taking into account the investigator's normal ranges. Listings of all variables will be provided by time and by treatment. Descriptive analysis will be presented by time and by treatment.

Physical examination

Listing of all variables will be provided by time and by treatment group. Descriptive analysis for raw data and for changes from baseline will be presented when appropriate.

Blood pressure and heart rate

Listing of all variables will be provided by time and by treatment. Descriptive analysis for raw data and for changes from baseline will be presented by time and by treatment.

ECG

Twelve-lead ECG will be recorded as described in Appendix I, at a speed of 25 mm/sec and calibrated at 10 mm/mV. If a QTc is prolonged (>450 ms or >60 ms vs. baseline), manual checking of QT and calculation of QTc with Bazett's formula will be performed by the investigator for safety purposes. When a patient will have completed treatment, all his ECGs will be transmitted to an experienced cardiologist for manual measurement as per Appendix J. The ECG parameters calculated manually by cardiologist will be used for statistical analysis.

Occurrence of infections

Information on distribution of infections to any bacterial strain will be collected and occurrence compared between study treatment.

11.4 Interim Analysis

No interim analysis is planned for this study. An assessment of the diagnosis of pneumonia will be confirmed by a panel of independent experts reviewing the clinical data under blinded conditions to ensure that a minimum of 10 documented pneumonia cases has been achieved.

11.5 Sample Size Justification

The sample size proposed is based on the primary clinical variable, ie. occurrence of pneumonia which is estimated at a 10% occurrence of pneumonia in colonized patients under mechanical ventilation. This reflects international clinical experience with patients under mechanical ventilation and respiratory tract colonization with *P. aeruginosa* (Expert Meeting, Paris, November 15, 2001). The majority of the recruited centres can provide historical figures between 10 and 15% occurence when no prevention therapy to *P. aeruginosa* is applied. This percentage however may fluctuate depending on country, hospital, department, patient subpopulations, season, and specific nosocomial strains at time of study.

The conservative figure of 10% has been elected which should correspond to recruiting 100 patients in the placebo study group for achieving 10 pneumonia cases. Depending on the degree of clinical efficacy

elicited by azithromycin treatment, the total patient figure may thus fluctuate between 200 assessable patients (10 pneumonia in placebo group, 0 pneumonia in active group, i.e. complete protection) and 100 patients (5 pneumonia in placebo group, 5 pneumonia in active group, i.e. no protection). This is based as described above on Fischer's exact test for the primary analysis variable, i.e. occurrence of pneumonia (two-sided, p=0.10, 10% in placebo group).

12. ETHICAL AND LEGAL ASPECTS

12.1 Good Clinical Practice

The procedures set out in this study protocol, pertaining to the conduct, evaluation, and documentation of this study, are designed to ensure that the sponsor and investigator abide by the principles of the good clinical practice (GCP), ICH guidelines, FDA or national guidelines as appropriate, and the ethical principles laid down in the current revision of the Declaration of Helsinki.

12.2 Delegation of Investigator Responsibilities

The investigator should ensure that all persons assisting with the trial are adequately informed about the protocol, any amendments to the protocol, the study treatments, and their trial-related duties and functions.

The investigator should maintain a list of subinvestigators and other appropriately qualified persons to whom he or she has delegated significant trial-related duties.

12.3 Subject Information and Informed Consent

Before being admitted to the clinical study, the subject must consent to participate after the nature, scope, and possible consequences of the clinical study have been explained in a form understandable to him or her. An informed consent document that includes both information about the study and the consent form will be prepared and given to the subject. This document will contain all elements required by national law and Sponsor’s requirements. The document must be in a language understandable to the subject and must specify who informed the subject. In majority of countries, the person who informs the subject must be a physician.

After reading the informed consent document, the subject must give consent in writing. The subject's consent must be confirmed at the time of consent by the personally dated signature of the subject and/or the subject's legally authorized representative such as a close family member in the event the patient is unable to follow or understand clearly the explanations provided and by the personally dated signature of the person conducting the informed consent discussions.

If the subject or the subject's legally authorized representative is unable to read, oral presentation and explanation of the written informed consent form and information to be supplied to subjects must take place in the presence of an impartial witness. Consent must be confirmed at the time of consent orally and by the personally dated signature of the subject or by the personally dated signature of the subject's legally authorized representative. The witness and the person conducting the informed consent discussions must also sign and personally date the consent document.

A copy of the signed consent document must be given to the subject or the subject's legally authorized representative. The original signed consent document will be retained by the investigator. In accordance with the ICH and national recommendations a copy of the signed informed consent document will be kept by the sponsor in a sealed envelope which will have been closed, identified, and signed on site by the investigator. This envelope can only be opened by the investigator or by the authorities at their request.

The investigator will not undertake any measures specifically required for the clinical study purposes until valid consent has been obtained.

12.4 Confidentiality

Subject names will not be supplied to the sponsor. Only the subject number and subject initials will be recorded in the CRF, and if the subject name appears on any other document (e.g. pathologist report), it must be obliterated before a copy of the document is supplied to the sponsor. Study findings stored on a computer will be filed in accordance with local data protection laws for computerized data. The subjects will be told that representatives of the sponsor, ERC or regulatory authorities may inspect their medical records to verify the information collected, and that all personal information made available for inspection will be handled in strictest confidence and in accordance with local data protection laws.

The investigator will maintain a personal subject identification list (subject numbers with the corresponding subject names) to enable records to be identified.

12.5 Protocol Amendments

Neither the investigator nor the sponsor's representatives will alter this study protocol without obtaining the written agreement of the other. Once the study has started, amendments should be made only in exceptional cases. The changes then become part of the study protocol.

12.6 Approval of the Study Protocol and Amendments

Before the start of the study, the study protocol, informed consent document, and any other appropriate documents will be submitted to the Ethical Review Committee (ERC) with a cover letter or a form listing the documents submitted, their dates of issue, and the site for which approval is sought. Before the study initiation, the sponsor will notify the authorities according to local legal requirements.

Study medication can only be supplied to the investigator after documentation on **all** ethical and legal requirements for starting the study has been received by the sponsor. This documentation must also include a list of the members of the ERC, including their gender, occupation, and qualifications. The opinion given by the ERC should specify the study title, study code, study site, amendment number if appropriate and any other documents reviewed. It must mention the date on which the decision was made and must be officially signed by a committee member, usually the president or secretary to the ERC. In the USA an FDA Form 1572 must be filled.

Before the first subject is enrolled in the study, all ethical and legal requirements must be met.

The ERC and, if applicable, the authorities must be informed of all subsequent protocol amendments, in accordance with the national legal requirements. Amendments must be evaluated to determine whether formal approval must be sought and whether the informed consent document should also be revised.

The investigator must keep a record of all communication with the ERC. This also applies to any communication between the investigator and the authorities.

12.7 Ongoing Information for the Ethical Review Committee (ERC)

If required by legislation or by the ERC, the investigator must submit to the ERC:

- Information on serious or unexpected adverse events as soon as possible
- Periodic reports on the progress of the study if it is requested on the initial reply of the ERC.

12.8 Premature Closure of the Study

The sponsor or the investigator has the right to close this study at any time. As far as possible, this should occur after mutual consultation. The ERC must be informed.

Should the study be closed prematurely, all study materials (completed, partially completed, and blank CRF, study medication, etc.) must be returned to the sponsor, as if the study had been completed.

12.9 Record Retention

The following records must be retained by the investigator until at least 2 years after the last approval of a marketing application in an ICH region and until there are no pending or contemplated marketing authorization in an ICH region, or at least 2 years have elapsed since the formal discontinuation of clinical devlopment of the investigational product. However, as a consequence of international regulatory requirements, the sponsor may request retention for a longer period. These essential documents include:

- Signed informed consent documents for all subjects
- Subject identification code list (must be kept 15 years according to European regulations), screening log (if applicable), and enrollment log
- Record of all communications between the investigator and the ERC
- Composition of the ERC
- Record of all communications between the investigator and sponsor's representatives
- List of subinvestigators and other appropriately qualified persons to whom the investigator has delegated significant trial-related duties, together with their roles and their signatures
- Copies of case report forms and of documentation of corrections for all subjects
- Drug accountability records
- Record of any body fluids or tissue samples retained
- All other source documents (patient records, hospital records, laboratory records, worksheets etc.)
- All other documents as listed in Section 8 of the ICH consolidated guideline on GCP (Essential Documents for the Conduct of a Clinical Trial) of by FDA Guidelines if appropriate (Form 1572 etc.)

However, because of international regulatory requirements, the sponsor may request retention for a longer period of time. The investigator must therefore obtain approval in writing from the sponsor prior to destruction of any records.

Normally, these records will be held in the investigator's archives. If the investigator is unable to meet this obligation, he must ask the sponsor for permission to make alternative arrangements. Details of these arrangements should be documented.

12.10 Liability and Insurance

In accordance with GCP and ICH guidelines, the sponsor has subscribed to an insurance policy covering, in its terms and provisions, its legal liability for injuries caused to participating persons and arising out of this research performed strictly in accordance with the scientific protocol as well as with applicable law and professional standards.

13. STUDY MONITORING AND AUDITING

Monitoring and auditing procedures developed by Quintiles will be followed, in order to comply with GCP guidelines. On-site checking of the case report forms for completeness and clarity, cross-checking with source documents, and clarification of administrative matters will be performed.

13.1 Study Monitoring

The study will be monitored by the sponsor’s representatives, by personal visits from the site monitor who will review the case report forms and source documents, supported on occasions by the study manager and clinical pharmacologist. By frequent communications (letter, telephone, and fax), the site monitor will ensure that the investigation is conducted according to protocol design and requirements, and to regulatory guidelines.

All unused study materials are to be returned to the sponsor after the clinical phase of the trial has been completed.

13.2 Source Data Verification and On-Site Audits

The national agency, the ERC, and/or a clinical quality assurance group delegated by the sponsor may request access to all source documents, case report forms, and other study documentation for on-site audit or inspection. Direct access to these documents must be guaranteed by the investigator, who must provide support at all times for these activities.

14. DOCUMENTATION AND USE OF STUDY FINDINGS

14.1 Documentation of Study Findings

A case report form (CRF) will be prepared by the investigator for each study part and carefully reviewed with the sponsor's representatives for adequacy and completeness of parameters listed. All protocol-required information collected during the study for each subject will be entered on the study worksheets or recorded as print outs or graphs (i.e. source documents). All clinical and safety data collected will be entered electronically by the investigator's designated representative, and printed CRF provided. Details of CRF completion and procedure for documentation of corrections will be agreed. Relevant PD data and PK data will be provided as tables. If the investigator authorizes other persons to make entries or corrections in the CRF, the names, positions, signatures, and initials of these persons must be supplied to the sponsor's representatives.

The investigator, or designated representative, should complete the worksheet pages as soon as possible after information is collected, preferably when a study subject is seen for an examination, treatment, or any other study procedure. Any outstanding entries must be completed immediately after the final examination. An explanation should be given for all missing data.

The site monitor will perform a 100 % accuracy check between source documents and CRF and printouts and do random and targeted checking of calculated parameters as estimated appropriate. A source data location list will be prepared and updated during the study. This list will be filed in both the trial master file and the investigator study file. The completed CRF as well as appropriate tables and graphs capturing raw and calculated data must be reviewed and signed by the investigator named in the study protocol or by a designated subinvestigator.

The site monitor will perform a 100 % accuracy check between source documents, tables, laboratory data, printouts etc. and CRF, also performing some random or targeted checking of calculated parameters as estimated appropriate.

The sponsor will retain the originals of all CRFs, 1 set of ECG copies for manual reading if needed, 1 set of ECG copies for archiving purposes, as well as copies of the source documents. The investigator will retain a copy of all CRFs, 1 set of ECG recording (original + copy), and the original source documents.

14.2 Use of Study Findings

All information concerning the product as well as any matter concerning the operation of Anbics AG, such as clinical indications for the drug and other scientific data relating to it, that have been provided by Anbics AG and are unpublished, are confidential and must remain the sole property of Anbics AG. The investigator will agree to use the information only for the purposes of carrying out this study and for no other purpose unless prior written permission from Anbics AG is obtained.

Anbics has full ownership of the original CRFs completed and other documents listed above as part of the study. By signing the study protocol, the investigator agrees that the results of the study may be used for the purposes of national and international registration, publication, and information for medical and pharmaceutical professionals. If necessary, the authorities will be notified of the investigator's name, address, qualifications, and extent of involvement.

The sponsor's representatives will prepare a final report on the study, with the support of the investigator who will perform the statistical and pharmacokinetic evaluation of the data. He will be required to sign a statement that he has read the report and confirms that, to the best of his knowledge, it accurately describes the conduct and results of the study.

The Coordinating Investigator, his delegate or Dr. C. van Delden shall have the right to present and to publish the scientific findings from the Study within a reasonable time after termination of the study and

availability of the clinical study report. Circulation of the manuscript among co-authors to achieve consensus should be performed in advance and the manuscript should be submitted to Anbics at least one month in advance for further discussion and approval. Co-authorship will reflect scientific involvement in study design, study performance, and/or analysis. Anbics is entitled to express any demand about the publication in order to protect its commercial interest. Requests and responses will be documented in writing.

15. STUDY DURATION AND DATES

The duration of this study is expected to be approximately 1 year, depending on total of patients recruited (range 120 to 240) to achieve 10 cases of pneumonia for a total between 100 and 200 assessable patients. A study start in November 2002 would enable completion in Autumn 2003 to achieve 200 assessable patients. The actual overall study duration or subject recruitment period may vary.

16. DECLARATIONS OF SPONSOR AND INVESTIGATOR

16.1 Declaration of Sponsor

This study protocol was subject to critical review and has been approved by the sponsor and its representatives. The information it contains is consistent with:

- The current risk-benefit evaluation of the investigational product
- The moral, ethical, and scientific principles governing clinical research as set out in the Declaration of Helsinki and the principles of GCP as described in the GCP and ICH guidelines.

The investigator will be supplied with details of any significance or new findings, including adverse events, relating to treatment with the investigational product.

Date:__________________

____
Clinical Pharmacologist's signature
Dr Françoise Brunner

16.2 Declaration of Investigator

I have received the following:

- The Package Insert (January 2001) which contain background information on clinical and non clinical data on azithromycin i.v. that is relevant to the study in human subjects
- The Investigator's Brochure dated October 2002

And I confirm that:

- I have been adequately informed about the scientific and medical rationale of the investigational product to date as described in the protocol introduction, including some relevant publications
- I have read this study protocol and agree that it contains all the information required to conduct the study
- I agree to conduct the study as set out in this protocol
- I will not enroll the first subject in the study until I have received approval from the appropriate ERC and until all legal requirements in my country have been fulfilled
- The study will be conducted in accordance with the moral, ethical, and scientific principles governing clinical research as set out in the Declaration of Helsinki, GCP and ICH Guidelines, and FDA guidelines if appropriate
- I agree to obtain, in the manner described in this study protocol, written informed consent or witnessed verbal informed consent to participate for all subjects enrolled in this study
- I am aware of the requirements for the correct reporting of serious adverse events, and I undertake to document and to report such events as requested
- I agree to supply the sponsor's representatives with evidence of current laboratory accreditation, the name and address of the laboratory, and a list of normal values and ranges
- I agree with the use of results of the study for the purposes of national and international registration, publication, and information for medical and pharmaceutical professionals
- I agree to keep all source documents and CRF as specified in Section *12.9 Record Retention* of this protocol
- I will provide a CV before the study starts, which may be submitted to regulatory authorities

Centre: _________________

____
City / Country

Date: __________________

____
Investigator's signature

Prof. ……………………….

Date: ____________________

____
Co-investigator's signature
Dr. ………………………….

Date: ____________________

____
Co-investigator's signature
Dr. ………………………….

Date: ____________________

____
Co-investigator's signature
Dr. ………………………….

17. REFERENCES

1. **Alvarez-Elcoro S and Enzler MJ**. 1999. The macrolides: erythromycin, clarithromycin, and azithromycin. Mayo Clinic Proc; **74**:613

2. **Baldwin DR, Wise R, Andrews JM, Ashby JP and Honeybourne D**. 1990. Azithromycin concentrations at the sites of pulmonary infection. Eur Respir J **3**: 886-890

3. **Davies DG, Parsek MR, Pearson JP, Iglewski BH, Costerton BH and Greenberg**. **EP.** 1998. The involvement of cell-to cell signals in the development of bacterial biofilm. Science **280**:295-298

4. **Dunne M, Fessel J, Kumar P, Dickensen G, Keiser P, Boulos M, Mogyros M, White AC Jr**., **Cahn P, O'Connor M, Lewi D, Green S, Tilles J, Hicks C, Bissett J and Schneider MME**. 2000. A randomized, double-blind trial comparing azithromycin and clarythromycin in the treatment of disseminated *Mycobacterium avium* infection in patients with human immunodeficiency virus. Clin Infect Dis **31**: 1245-1252

5. **Fujii T, Kadota J, Kawakami K, Iida K, Shirai R, Kaseda M, Kawamoto S and Kohno S.** 1995. Long term effect of erythromycin therapy in patients with chronic *Pseudomonas aeruginosa* infection. Thorax **50**: 1246-1252

6. **Garey KW and Amsden GW.** 1999. Intravenous azithromycin. Ann Pharmacother **33:** 218-228

7. **Gupta S Leatham EW, Carrington D, Mendall MA, Kaski JC and Camm AJ.** 1997. Elevated *Chlamidia pneumoniae* antibodies, cardiovascular events, and azithromycin in male survivors of myocardial infarction. Circulation **96**: 404-407

8. **Howe RA and Spencer RC.** 1997. Macrolides for the treatment of *Pseudomonas aeruginosa* infections? J Antimicrobial Chemotherapy **40**: 153-155

9. **Ichimiya T, Yamasaki T and Nasu M.** 1994. In vitro effects of antimicrobial agents on *Pseudomonas aeruginosa* biofilm formation. J Antimicob Chemotherapy **34**: 331-341

10. **Ichimiya T, Takeoka K, Hiramatsu K, Hirai K, Yamasaki T and Nasu M.** 1996. The influence of azithromycin on the biofilm formation of *Pseudomonas aeruginosa* in vitro. Chemotherapy **42**: 186-191

11. **Jackson LA, Stewart DK, Wang SP, Cooke DB, Cantrell T and Grayston JT.** 1999. Safety and effect on anti-Chlamydia antibody titres of a 1 month course study of daily azithromycin in adults with coronary disease. J Antimicrob Chemother **44**: 411-414

12. **Jaffe A, Francis J, Rosenthal M and Bush A**. 1998. Long-term azithromycin may improve lung function in children with cystic fibrosis. Lancet **351**:420

13. **Knaus WA, Draper EA, Wagner DP and Zimmermann JE**. 1985. APACHE II: a severity of disease classification system. Crit Care Med **13**:818-829

14. **Koletar SL, Berry AJ, Cynamon MH, Jacobson J, Currier JS, MacGregor RR, Dunne MW and Williams DJ**. 1999. Azithromycin as treatment for disseminated *Mycobacterium avium* complex in AIDS patients. Antimicrob Agents Chemother **43**: 2869-2872

15. **Luke DR, Foulds G, Cohen SF and Levy B**. 1996. Safety, toleration, and pharmacokinetics of intravenous azithromycin. Antimicrob Agents Chemother **40**: 2577-2581

16. **Nicolau DP, Banevicius MA, Nightingale CH and Quintiliani R**. 1999. Beneficial effect of adjunctive azithromycin in treatment of mucoid *Pseudomonas aeruginosa* pneumonia in the murine model. Antimicrob Agents Chemother **43**:3033-3035

17. **Olsen KM, San Pedro GF, Gann LP, Gubbins PO, Haliski DM, Campbell DC**. 1996. Intrapulmonary pharmacokinetics of azithromycin in healthy volunteers given five oral doses. Antimicrob Agents Chemother **40**: 2582-2585

18. **Peckham DG**. 2002. Macrolide antibiotics and cystic fibrosis. Thorax **57**: 189-190

19. **Piscitelli SC, Danziger LH, Rodvold KA**. 1992. Clarithromycin and azithromycin: new macrolide antibiotics. Clin Pharmacol Ther **11**: 137

20. **Pugh RNH, Murray-Lyon LM, Dawson JL, Pietronic MC and Williams R.** 1973. Transection of the esophagus for bleeding esophageal varices. Br J Surg **60**:646-649

21. **Pugin JR, Auckenthaler R, Mili N, Janssens JP, Lew RD and Suter PM**. 1991. Diagnosis of ventilator-associated pneumonia by bacteriologic analysis of bronchoscopic and non bronchoscopic „blind“ bronchoalveolar lavage fluid. Am Rev Respir Dis **143**:1121-1129

22. **Rapp RP**. 1998. Pharmacokinetics and Pharmacodynamics of Intravenous and Oral Azithromycin: enhanced tissue activity and minimal drug interactions. Ann Pharmacother **32**: 785-793

23. **Reese RE and Betts RF**. 1996. Erythromycin, azithromycin, clarithromycin, and dirithromycin. In: Reese RE, Betts RF, eds. A practical approach to infectious diseases, 4th ed. Boston: Little, Brown, 1996: Chap. 28M

24. **Rumbaugh KP, Griswold JA and Hamood AN**. 2000. The role of quorum sensing in the *in vivo* virulence of *Pseudomonas aeruginosa*. Microbes. Infect **2**:1721-1731

25. **Schentag JJ and Ballow CH**. 1991.Tissue directed pharmacokinetics. Am J Med **91**: 5S (suppl 3A)

26. **Singh N, Rogers P, Atwood CW, Wagener MM and Yu VL.** 2000. Short-course empiric antibiotic therapy for patients with pulmonary infiltrates in the intense care unit. Am J Respir Crit Care Med **162**:505-511

27. **Sorvillo F, Beall G, Turner PA, Beer VL, Kovacs AA and Kerndt PR.** 2001. Incidence and determinants of *Pseudomonas aeruginosa* infection among persons with HIV: association with hospital exposure. Am J Infec Control **29**: 79-84

28. **Tateda K, Ishii Y, Matsumoto T, Furuya N, Nagashima M, Matsunaga T, Ohno A, Miyazaki S and Yamaguchi K** 1996. Direct evidence for antipseudomonal activity of macrolides; Exposure-dependent bactericidal activity and inhiition of protein synthesis by erythromycin, clarithromycin, and azithromycin. Antimicrob Agents Chemother **40**:2271-2275

29. **Tateda K, Comte R, Pechere JC, Kohler T, Yamaguchi K and Van Delden C**. 2001. Azithromycin inhibits quorum sensing in *Pseudomonas aeruginosa*. Antimicrob Agents Chemother **45**:1930-1933

30. **Van Delden C and Iglewski BH**. 1998. Cell-to-Cell signaling and *Pseudomonas aeruginosa* infections. Emerging Infectious Diseases **4**:551-560

31. **Walter J, Seeney S, Bell S, Bowler S, Masel P and MacCormack J**. 2002. Effect of log term treatment with azithromycin on disease parameters in cystic fibrosis: a randomized trial. Thorax **57**: 212-216

32. **Ward TT, Rimland D, Kauffman C, Huycke M, Evans TG and Heifets**. 1998. Randomized, open-label trial of azithromycin plus ethambutol vs. clarithromycin plus ethambutol as therapy for Mycobacterium avium complex bacteremia in patients with human immunodeficiency virus infection. Clin Infect Dis **27**: 1278-1285

33. **Zithromax® Package Insert** (azithromycin for injection) by Pfizer Inc, issued in January 2001

APPENDIX A

Child Pugh's Score

Clinical and Laboratory Signs	Scoring* by Anomaly		
	**1**	**2**	**3**
**Hepatic encephalopathy (stage)**	Absent	1 or 2	3 or 4
**Ascites**	Absent	Moderate	Abundant
**Bilirubin** (µmol/L**)**	17 - 34	35 - 51	> 51
**Albuminemia** (g/L)	> 35	28 - 35	< 28
**Prothrombin time** (% of normal)<br>**Prolongation vs. control** (seconds)	> 70<br>< 4	40 - 70<br>4 – 6	< 40<br>>6

Score by the sum of the points for each sign:

**Grade A**	5 to 6
**Grade B**	7 to 9
**Grade C**	10 to 15

---

* **Pugh RNH, Murray**-Lyon LM, Dawson JL, Pietronic MC and Williams R. 1973. Transection of the esophagus for bleeding esophageal varices. Br J Surg **60**:646-649

APPENDIX B

Laboratory Safety

Hematology
Hemoglobin total
Hematocrit
RBC
Mean corpuscular hemoglobin (MCH)
Mean corpuscular volume (MCV)
WBC with differential count (neutrophils, lymphocates, monocytes, eosinophils, basophils)
Platelet count
Prothrombin time (PT)
Activated partial thromboplastin time (aPTT)

Blood chemistry
Fasting glucose
Albumin, total protein
Creatinine
Urea
Sodium, potassium, calcium, chloride
Uric acid
AST, ALT, CPK, AP,
Gamma glutamyl transferase
Total bilirubin

Urinalysis

Macroscopic examination (dipstick) at screening and study completion (Day n or Day 21)
pH
Protein
Glucose
Ketones
Leucocytes
Blood
Urobilinogen
Bilirubin

APPENDIX C

Preparation of Drug Treatment Solutions for i.v. Administration

Material

Sterile pouches containing 250 mL of sterile saline (0.9 % sodium chloride)

Syringes 5 mL

Twin set of labels printed by Quintiles Clinical Supplies (left side to be pasted onto pouch by pharmacist, right side to be torn off by investigator prior to infusion and pasted on CRF)

Pharmacist

The pharmacist will prepare the supplies using the patient schedule provided by the investigator, the patient allocation number and the randomization list provided by the statistical department.

A. For patients allocated to treatment with azithromycin

- Aspirate 4.8 mL sterile water for injection with a 5 mL syringe and add to the lyophilized azithromycin vial
- Shake each vial gently until complete dissolution has taken place (100 mg/mL)
- Check that no particulate matters are left by carefully mirroring the vial against light
- Withdraw with a syringe 3.0 mL from a pouch containing 250 mL of sterile saline and replace by 3.0 mL of reconstituted azithromycin solution at 100 mg/mL solution, leading to a final 250 mL solution at 1.2 mg/mL
- Attach the appropriate study label (left side) with study number, study day and patient allocation number
- Transfer 1, 3 or 6 infusion pouches to the investigator as jointly agreed to cover the needs for 1, 3, or 6 days of treatment, along with the additional labels to be attached onto the CRFs
- Keep all used azithromycin vials to enable compliance monitoring

B. For patients allocated to treatment with placebo

- Use a pouch containing 250 mL of sterile saline
- Attach the appropriate study label (left side) with study number, study day and patient allocation number to each pouch
- Transfer 1, 3 or 6 infusion pouches to the investigator as jointly agreed, along with additional labels to be attached onto the CRFs

Investigator:

- Store the transmitted pouches at room temperature (<30 °C or >86 °F) if for 24 hours, else at 5 °C or 41 °F) for a maximum of 6 days
- If maintained refrigerated, take out the appropriate pouch at least 1 hour prior to drug administration
- Tear off the right part of the label and attach onto the CRF
- Suspend the pouch and connecting line onto the peristaltic pump for a delivery rate of 250 mL / hour
- Record carefully time of infusion start (t=0) and completion (1 hour), ultimately discarding the pouch

APPENDIX D

Collection and Storage of Tracheal Aspirates

Material

- Sterile catheter for endoscopy
- Special double walled tubes for microbiology 50 mL (type, company, city, country) with screw caps for storage and transport of infectious specimens
- Labels to be printed by Quintiles

Collection of tracheal aspirates

1. Several tracheal aspirates will be performed daily as often as medically indicated to ensure reasonable ventilation, using the routine, standardized procedure of the ICU and maintaining as aseptic conditions as possible during aspiration, collection and transfer of aspirate specimens to the labeled storage tube
2. Apply 100% O_2 bagging just before suctioning for collecting tracheal aspirates through the tracheal tube
3. **For aspirate 1 and 2, avoid whenever possible to dilute samples and no not use saline prior to collection to facilitate suction of viscous material**
4. For other aspirates, inject appropriate amount of saline into the tube to facilitate suction as per study site routine. As much as possible this amount should be kept stable for a given patient throughout the study and the precise volume recorded in the CRF
5. Insert the catheter at full-length and withdraw slowly with suction applied intermittently according to routine at ICU
6. Collect the tracheal aspirates in the prelabelled tube(s)
7. Estimate total volume and record in the CRF
8. For the 2 daily aspirates, the exact time should be recorded in the CRF
9. The first specimen of the day (e.g. Day 3, #1) will be collected by the ICU nurse after awaking, prior to administering azithromycin or placebo (0 hour specimen) and will be used for local bacteriology experiments (daily for isolation of *P. aeruginosa* strain, every third day for quantification as recommended in Study Schedule and in Sections 7.2.2 to 7.2.5) Strains will be stored at –70°C and shipped to Geneva for *in vitro* experiments
10. The second specimen of the day (e.g. Day 3, #2) will be collected daily by the ICU nurse early afternoon (ideally ~6 hours after infusion start), transferred to –70°C freezer at earlier possible time and will be shipped to Geneva for *in vivo* experiments performed in Geneva's laboratory as recommended in Study Schedule and in Sections 7.2.2 to 7.2.5 or stored as reserve samples
11. The total number of aspiration procedures per day and total volume thus collected will be recorded in the CRF as well as saline volume if appropriate

Specifications for tube labels

1. Labels for isolated strains (from Aspirate #1) or for tracheal aspirates (from Aspirate #2) will contain the following information: ANB 006 #2001, subject allocation number No, study day (Day 1 to 21), matrix (P. aeruginosa; trach asp. sample number (1, 2), sampling time, and date
2. One label will be attached to the internal tube (#1, 2), and one to the external tube(#1, 2)

APPENDIX D

Collection and Storage of Tracheal Aspirates (cont’d)

Storage

1. For specimens #1, store at 5°C (41 °F) and transmit daily to local laboratory (see Study Schedule and Section 7.2.) for strain isolation and quantification (usually every 3rd day)
2. Deep freeze at –70°C isolated *P. aeruginosa* strains on skim milk and glycerol otained from Aspirate 1
3. Deep freeze specimens #2 (Geneva experiments) at –70 °C as quickly as possible, pending shipment in batches as agreed with study monitor. The parameters measured are listed under Study Schedule and Section 7.3.

APPENDIX E

Collection and Assessment of Bronchial Alveolar Lavage (BAL),

Bronchiotracheal Aspirate or Protected Brush Specimen (PBS) on Day x

Material

- Sterile fibroscope tip or sterile protected specimen brush
- Sterile catheter
- Sterile saline (0.9 % sodium chloride)
- Special double walled tubes for microbiology 200 mL of adequate size, with screw caps for storage and transport of infectious specimens
- Labels to be printed by Quintiles

Collection of BAL (or bronchotracheal aspirate or PBS)

1. Depending on clinic setup, ICU routine, and patient condition, 3 different techniques may be used on the presumed Day x for confirming diagnosis of pneumonia:
    - Bronchoalveolar lavage (BAL)
    - Bronchotracheal aspirate
    - Protected brush specimen (PBS)
2. A BAL specimen will be taken only if there is strong suspicion of pneumonia to confirm diagnosis (Day x). This procedure should be performed by a trained intense care physician, only if medically not contraindicated, under aseptic conditions and operating as fast as possible to minimize the period when the patient is weaned off from mechanical ventilation. The procedure must be completed fast, using the routine, standardized procedure of the ICU and maintaining as aseptic conditions as possible during aspiration, collection and transfer of BAL specimens to the labeled storage tubes
3. Apply 100% O_2 bagging technique just before shifting the fibroscope or the brush through the tracheal tube
4. The fibroscope tip or brush will be positioned in a segmental ostium (lingual or right middle lobe), the selected side depending on the opacities seen on the lung radiography
5. In case of BAL, 2 aliquots of 50 mL of tepid saline will be infused through the aspiration port and collected via the same port by suction to collect the BAL fluid in 2 sterile tubes:
    - 1 tube will be sent to local laboratory for CFU analysis for diagnostic purposes, centrifugation, and differential cytology
    - 1 tube will be stored at –70 °C pending shipment to Geneva for special analysis (see Section 7.2.3)
6. If the protected brush technique is used, the brush will be disengaged from its sheath when *in situ,* gently sweeping the bronchial wall upon withdrawing. This specimen will be kept for local laboratory assessment. No specimen for the Geneva laboratory will be available using this technique
7. The patient will be put immediately back onto the mechanical ventilation as soon as the procedure is completed

APPENDIX E

Collection and Assessment of Bronchial Alveolar Lavage (BAL),

Bronchiotracheal Aspirate or Protected Brush Specimen (PBS) on Day x (cont'd)

Specifications for tube labels

1. Labels for tracheal aspirates will contain the following information: ANB 006 #2001, subject allocation number No, study day (Day x), matrix (BAL, brush, bronchial aspirate), sampling time, and date
2. One label will be attached to the internal tube and one to the external tube if appropriate (shipment to Geneva)

APPENDIX F

Recovery and Storage of Endotracheal Device

Material

- Sterile endotracheal tube, with T- tube and cuff
- Sterile bag
- Large container for microbiology of adequate size, with screw caps for storage and transport of infectious specimens
- Labels to be printed by Quintiles

Recovery of endotracheal device

1. On Day n –1 (i.e. on day of extubation or of exitus, but usually not if Day 20 unless ventilation may actually be discontinued in that given patient after 20 days of treatment), carefully remove the complete endotracheal device as aseptically as possible
2. Transfer into an appropriate sterile bag which will be sealed and freeze at –70°C as soon as possible
3. Pack into an appropriate container with screw lid when required for transport
4. In the event 2 endotrachal tubes have been used for a given patient (i.e. if the first extubation was not successful and the patient had to be re-intubated within 24 hours), one or two endotracheal tubes may be collected and transferred to Geneva laboratory

Specifications for tube labels

1. Labels for tracheal tubes will contain the following information: ANB 006 #2001, subject allocation number No, study day (Day n-1), tracheal tube, recovery time time, and date
2. One label will be attached to the sterile bag and one to the container as appropriate (shipment to Geneva)

APPENDIX G

Transport of Biological Specimens & Endotracheal Device

The packing will be the responsibility of the site monitor or delegate. Shipments will be organized in each centre in batches with specimens from 4 to 8 patients, using dedicated card boxes for each patient and matrix, with samples organized in a chronological order as per attached grid on box lid

- Do not allow any specimen samples to thaw at any time during the shipping preparation process
- Pack daily *P. aeruginosa* strains (obtained from Aspirate #1) and daily tracheal aspirate samples #2, BAL from Day x if available, and the intubation device collected on Day n-1 in chronological order, by patient, in large divided cardboard boxes to ensure protection against mechanical damage during shipment
- Seal carefully each cardboard box and record on each box study code, patient number and samples
- Include in the insulated container a photocopy of sample collection dates and time for each patient
- Place dry ice plates at bottom, sides and top of each insulated box, forming a house around the cardboard boxes containing the specimens and ensuring ~72 hours of deep freezing
- Close each insulated box with extra large tape and special plastic strings
- Apply on each insulated box the appropriate address and shipment labels (for dry ice, for bacteriological material etc.) as required for air freight
- Prepare the required proforma invoice and affix on each insulated box
- Ship the insulated boxes, preferably on Monday or Tuesday to:

  Dr. Christian van Delden
  Département de Génétique et Microbiologie
  Centre Médical Universitaire
  1, rue Michel-Servet
  CH-1211 Geneva
  Switzerland
  Tel: xx 41 22 702 5639
  Fax: xx 41 22 702 5702

  To the attention of a laboratory technician to be specifically nominated

- Prior to each shipment and at least 3 days in advance forward a Fax message to the laboratory technician indicating the date of arrival in Geneva, flight, airway bill number, study number, study site, patient allocation numbers, amount of samples being shipped, number of boxes, a model of the proforma invoice, and a list of samples
- Obtain confirmation of availability by Fax and phone prior to shipment ensuring that the Geneva laboratory will be available to receive and store the samples on the projected delivery day
- Upon arrival in Geneva, the samples should be stored at –70 °C, pending investigations listed in Section 7.2.2

APPENDIX H

Acute Physiology Chronic Health Evaluation (APACHE II)

**Physiological Variable**	**High Abnormal Range**					**Low Abnormal Range**			
	**+4**	**+3**	**+2**	**+1**	**0**	**+1**	**+2**	**+3**	**+4**
Temperature (rectal, °C)	☐<br>≥41.0°	☐<br>39.0°-40.9°		☐<br>38.5°-38.9°	☐<br>36.0°-38.4°	☐<br>34.0-35.9°	☐<br>32.0°-33.9°	☐<br>30.0°-31.9°	☐<br>≤29.9°
Mean blood pressure (mm Hg)	☐ ≥160	☐ 130-159	☐ 110-129		☐ 70-109		☐ 50-69		☐ ≤49
Heart rate (ventricular)	☐ ≥180	☐ 140-179	☐ 110-139		☐ 70-109		☐ 55-69	☐ 40-54	☐ ≤39
Respiration rate (ventilated or not)	☐ ≥50	☐ 35-49		☐ 25-34	☐ 12-24	☐ 10-11	☐ 6-9		☐ ≤5
Oxygenation: A-aD0$_2$ or Pa$_{O2}$ (mm Hg)<br>a. FI$_{O2}$ ≥0.5 record A-aD0$_2$	☐<br>≥500	☐<br>350-499	☐<br>200-349		☐<br><200				
b. FI$_{O2}$ <0.5 record only PaO$_2$					☐ PaO$_2$<br>>70	☐ PaO$_2$<br>61-70		☐ PaO$_2$<br>55-60	☐ PaO$_2$<br><55
Arterial pH	☐ ≥7.7	☐ 7.6-7.69		☐ 7.5-7.59	☐ 7.33-7.49		☐7.25-7.32	☐7.15-7.24	☐ <7.15
Serum sodium (mmol/L)	☐ ≥180	☐ 160-179	☐ 155-159	☐ 150-154	☐ 130-149		☐ 120-129	☐ 111-119	☐ ≤110
Serum potassium (mmol/L)	☐ ≥7.0	☐ 6-6.9		☐ 5.5-5.9	☐ 3.5-5.4	☐ 3-3.4	☐ 2.5-2.9		☐ <2.5
Serum creatinine (μmol/L)<br>**Acute renal failure: double score**	☐ ≥3.1<br>☐	☐ 1.8-3.0<br>☐	☐ 1.3-1.7<br>☐		☐ 0.5-1.2<br>☐		☐ <0.5<br>☐		
Hematocrit (%)	☐ ≥60		☐ 50-59.9	☐ 46-49.9	☐ 30-45.9		☐ 20-29.9		☐ <20
WBC (ths/mm^3)	☐ ≥40		☐ 20-39.9	☐ 15-19.9	☐ 3-14.9		☐ 1-2.9		☐ <1
Glasgow Coma Score (GCS)<br>Score = 15 minus actual GCS									
**A** **Acute Physiology Score (APS): Sum of 12 variables**									
Serum HCO$_3$ (venous mmol/L)<br>Not preferred, only if no arterial pH	☐ ≥52	☐ 41-51.9		☐ 32-40.9	☐ 22-31.9		☐ 18-21.9	☐ 15-17.9	☐ <15

APPENDIX H

Acute Physiology Chronic Health Evaluation (APACHE II) (cont'd)

B :   Age Points

**B**	**Age (years)**	**≤44**	**45-54**	**55-64**	**65-74**	**≥75**
	**Assign Points**	☐ 0	☐ 2	☐ 3	☐ 5	☐ 6

C :   Chronic Health Points

**C**	**If the patient has a history of severe organ system insufficiency or is immuno-compromised prior to hospital administration***	**Assign Points**
	a. For nonoperative or emergency postoperative patients	☐ 5
	b. For elective postoperative patients	☐ 2

*** Definitions**

Liver: Biopsy proven cirrhosis and documented portal hypertension; episodes of upper GI bleeding attributed to portal hypertension; prior episodes of hepatic failure/encephalopathy/coma

Cardiovascular: New York Heart Association Class IV

Respiratory: Chronic restrictive, obstructive or vascular disease resulting in severe exercise restriction; documented chronic hypoxia, hypercapnia, secondary polycythemia, severe pulmonary hypertension (>40 mm Hg) or respiratory dependency

Renal: Receiving chronic dialysis

Immuno-compromised: Drug-induced by recent and high dose steroid, chemotherapy, radiation, cyclosporine…
Disease-induced in leukemia, lymphoma, or AIDS

**APACHE II**		**Score**
**APS Points**	**A**	
**Age Points**	**B**	
**Chronic Health Points**	**C**	
**Total APACHE II Score**		

Maximum possible score :	71
Maximum clinical score:	55
**Range for selection:**	**10 to 25**

APPENDIX I

Clinical Pulmonary Infection Score (CPIS)

This Clinical Pulmonary Infection Score (CPIS) has been originally developed by Pugin et al (1991), and later adapted by Singh et al (2000) to diagnose pneumonia on the basis of 7 parameters listed in the table below with the appropriate scoring system (for details on measurements, see Study Schedule on page 10 and Section 7.1.2):

Item	Units	Score 0	Score 1	Score 2
Body temperature	°C	≥36.5 and ≤38.4	≥38.5 and ≤38.9	≥39.0 or ≤36.0
Blood leukocytes	mm3	≥4'000 and ≤11'000	<4'000 and >11'000	<4'000 and >11'000 and band forms ≥ 50%
Tracheal secretions		Absent	Present Non purulent	Present Purulent
Oxygenation Pa $_{O2}$/Fi$_{O2}$	mm Hg	>240 or ARDS*	--	≤240 and no ARDS*
Lung X-ray infiltrate		Absent	Diffuse or patchy	Localized
Progression of lung infiltrate		No progression	--	Progression (after CHF and ARDS excluded)
Tracheal aspirate culture (predominant pathogen)		None or rare or +	Moderate or heavy in culture	Same organism on Gram stain

ARDS = Acute respiratory distress syndrome

Defined as Pa $_{O2}$/Fi$_{O2}$ ≤200, pulmonary wedge pressure ≤18, and acute pulmonary infiltrates

CHF = Congestive heart failure

Patients with a total CPIS for *Pseudomonas aeruginosa* of:

- **<6 are unlikely to have pneumonia**
- **≥6 have pneumonia**
  - **IF BAL ≥10^4 CFU/mL and possible pneumonia if lower**
  - **OR if bronchotracheal lavage ≥10^5 CFU/mL and possible pneumonia if lower**
  - **OR if protected specimen brush ≥10^3 CFU/mL and possible pneumonia if lower**

APPENDIX J

Electrocardiogram (ECG)

Recording

- Record 12-lead ECGs at a speed of 25 mm/sec using a validated electrocardiograph calibrated to 10 mm/mV (at least 5 complexes of good quality, 1 specimen at each time point as source document)
- Copies of original will be made for archiving purposes (1 set for investigator, 1 set for sponsor)
- Ensure that the subject has been supine for at least 10 minutes without disturbance such as aspiration prior to recording the ECG, avoiding event or activity known to prolong QT (falling asleep etc.)

Automated Analysis

- Cardiac frequency, PR interval, QT, QTc with Bazett's formula, QRS duration, possible U wave will be collected for safety purposes but will not be recorded in the CRF
- If QTc is prolonged (>450 msec) or if an artefact with the ECG machine reading is suspected, the QT will be measured manually by the investigator and QTc corrected with Bazett's formula

Assessment on Manual Parameters

- At study completion, transmit all ECGs for a given patient to an experienced cardiologist at each centre
- All ECG parameters will be measured manually by this cardiologist according to standard practice (HR, RR, QT)
- QT calculated for QTc (Bazett's) and wave morphology analysed for assessment at different time points vs. their respective pretreatment baseline (Δ)
- These data will be part of the CRF and of the Clinical Study Report
- Unless required at later stage, no centralized analysis is planned
